# A model-based assessment of anthropogenic disturbance on lotic macroinvertebrate assemblages

**DOI:** 10.1002/ecs2.70361

**Published:** 2025-10-05

**Authors:** Darin A. Kopp, John L. Stoddard, Philip R. Kaufmann, Alan T. Herlihy, Ryan A. Hill, Meredith M. Brehob, Robert D. Sabo

**Affiliations:** 1 United States Environmental Protection Agency, Office of Research and Development, Center for Public Health and Environmental Assessment, Corvallis, Oregon, USA; 2 Department of Fisheries, Wildlife, & Conservation Sciences, Oregon State University, Corvallis, Oregon, USA; 3 Oak Ridge Institute for Science and Education, Participant c/o US EPA, ORD, Washington, DC, USA; 4 United States Environmental Protection Agency, Office of Research and Development, Center for Public Health and Environmental Assessment, Washington, DC, USA

**Keywords:** benthic macroinvertebrate, joint species distribution modeling, National Aquatic Resource Surveys, National Rivers and Streams Assessment, reference condition, richness

## Abstract

Traditionally, the effects of anthropogenic disturbance on biological assemblages are elucidated by comparing an assemblage observed at a site to one that represents a minimally disturbed state. Unfortunately, defining a minimally disturbed state is extremely challenging because of the extent of human disturbance. We use a national scale dataset and a model-based approach to assess how benthic macroinvertebrate assemblages at 1748 sites would change if common anthropogenic disturbances were removed from in-stream physiochemical variables. First, we used random forest models and current landscape data to predict physiochemical conditions and then infer abiotic condition in the absence of disturbance. Second, we combined these estimates with joint species distribution models to predict the assemblage that could occur in these undisturbed conditions. Random forest models explained 48%–75% of the variation in log-transformed total nitrogen, phosphorus, sulfate, chloride, and substrate diameter. Generally, nutrient and salinity concentrations were higher, and substrates were finer than predicted to be without disturbances. Using these physiochemical data, joint species distribution models, fitted to nine separate ecoregions, accurately explained genus richness (*R*^2^ = 0.73–0.85) and composition (Jaccard similarity index = 0.48–0.55). Depending on the ecoregion, we found that genus richness could increase or decrease at 26%–61% of sites if disturbance was removed. For example, with anthropogenic disturbance, occurrence probabilities for Ephemeroptera, Plecoptera, and Trichoptera decreased at 5%–26% of sites, while occurrence probabilities for Mollusca and other noninsect, non-arthropod taxa increased at 5%–33% and 11%–24% of sites, respectively. Importantly, our innovative framework provides a foundation for evaluating the effects of anthropogenic disturbance on macroinvertebrate assemblages without identifying reference sites.

## INTRODUCTION

Quantifying the extent and magnitude of anthropogenic disturbance on ecosystems requires a benchmark that represents a desired, expected, or previous condition (Hawkins et al., 2010; McNellie et al., 2020; Stoddard et al., 2006). Ideally, benchmarks should represent chemical, physical, and biological characteristics with minimal anthropogenic disturbance (Reynoldson et al., 1997; Stoddard et al., 2006). However, for many ecosystems, the minimally disturbed condition is difficult to quantify or may no longer exist (Dudgeon et al., 2006; McNellie et al., 2020; Vörösmarty et al., 2010). The concept of “least disturbed” describes conditions that are derived from a collection of sites that represent the best available, given today’s landscape (Stoddard et al., 2006). Unfortunately, sites representing the best available can be spatially aggregated and vary in quality because anthropogenic disturbance is not evenly distributed across the landscape (Herlihy et al., 2008; McNellie et al., 2020; Yuan et al., 2024). If least-disturbed sites are spatially aggregated, their ecological, chemical, and physical conditions may be unattainable for some locations because of differences in natural setting or biogeographic history. Alternatively, if reference site quality is lower (i.e., defined differently) for some ecosystems because of a relatively long legacy of human intensification, some sites will be judged against a lower benchmark and erroneously appear to be in better condition than others. Although least-disturbed reference sites provide reasonable and defensible benchmarks for assessing biological condition (Herlihy et al., 2008; Mitchell et al., 2025; Stoddard et al., 2006), they are typically not undisturbed, nor evenly distributed among all ecosystem types (McNellie et al., 2020; Yuan et al., 2024). Novel approaches and concepts are urgently needed to overcome these limitations.

Using empirical models to quantify taxon–environment relationships may provide an alternative to relying on reference sites because they can elucidate how individual taxa and entire assemblages change along physiochemical gradients (Chessman & Royal, 2004; Elias et al., 2016; Kilgour & Stanfield, 2006; Kopp et al., 2023). Anthropogenic disturbance typically manifests as altered physiochemical conditions (Fergus et al., 2023; Tang et al., 2020) such that quantifying taxon–environment relationships across space could elucidate how organisms may change with disturbance across time (Blüthgen et al., 2022). For example, taxa that have a negative relationship along an environmental gradient could be expected to become less prevalent if human disturbance contributes to more extreme environmental conditions and more prevalent if reducing human disturbance contributes to less extreme conditions (Kopp et al., 2023). Further, these relationships may be useful for biological assessments because they can predict the presence of taxa at a site if the physiochemical environment was not disturbed and compared to the taxa that were actually observed (Chessman & Royal, 2004; Elias et al., 2016). If the observed assemblage is similar to the predicted assemblage, then the site may be considered unaffected by anthropogenic disturbances.

Species distribution modeling has advanced our ability to quantify taxon–environment relationships (Franklin, 2010; Guisan et al., 2017; Ovaskainen & Abrego, 2020). Species distribution modeling consists of a diverse set of methods that typically focus on quantifying taxon–environment relationships for individual species (Elith & Leathwick, 2009). Joint species distribution modeling (JSDM) is a recent multivariate extension of single species distribution modeling, capable of estimating taxon–environment relationships for all members of an assemblage simultaneously. Because of their complex structure, JSDMs are often fitted using Bayesian inference. As such, the resulting posterior distribution can be used for hypothesis testing (Johnson et al., 2022). For example, given a posterior distribution of values representing a biological assemblage (e.g., taxonomic richness) in the absence of human disturbance, it is possible to evaluate whether an observed assemblage is consistent with this distribution. In addition, these models use latent factors to account for unmeasured environmental variables and potential biotic interactions (Ovaskainen & Abrego, 2020; Ovaskainen et al., 2017; Warton et al., 2015). This feature is particularly important for biological assessments because it can strengthen the inference gained from evaluating a specific suite of environmental variables that are typically altered by human disturbance. Thus, the ability to model all taxa in an assemblage and probabilistically evaluate whether an observation is consistent with a posterior distribution makes JSDMs well suited for understanding how anthropogenic factors affect biological assemblages.

Although taxon–environment relationships are necessary to circumvent the need for reference sites, they require a concurrent expectation of physiochemical conditions if anthropogenic disturbances were removed or reduced. Traditionally, undisturbed physiochemical conditions are obtained or modeled from least-disturbed reference sites (Olson & Cormier, 2019; Olson & Hawkins, 2012). However, empirical models have also linked changes in the physiochemical environment to anthropogenic activities and been used to estimate conditions if anthropogenic disturbance is reduced or eliminated without relying on reference sites (Dodds & Oakes, 2004; Herlihy & Sifneos, 2008; Soranno et al., 2011). Random forest models are a machine learning algorithm that have superior predictive performance than other modeling techniques (Peters et al., 2007; Prasad et al., 2006) and are often used to model water quality parameters and physical habitat characteristics (Olson & Cormier, 2019; Olson & Hawkins, 2013; Sabo et al., 2023; Yuan et al., 2024). Further, these models are more robust to nonlinearities, multicollinearity, and overfitting (Breiman, 2001) and can evaluate the relative importance of different predictor variables (Lin et al., 2021; Sabo et al., 2023). Because these algorithms can accommodate complex relationships and a relatively high number of covariates, they are well suited for evaluating the relative importance of natural and anthropogenic variables on physiochemical conditions and, in turn, predicting the abiotic conditions if anthropogenic disturbances were removed, provided the new data are sufficiently similar (not extrapolated) to the training dataset (Meyer & Pebesma, 2021; Yuan et al., 2024). Indeed, efforts that combined estimates of undisturbed physiochemical conditions with biological models have made progress toward addressing the limitations of reference site-based approaches (Chessman & Royal, 2004; Elias et al., 2016; Kilgour & Stanfield, 2006; Yuan et al., 2024).

In rivers and streams, biological condition assessments often rely on surveys of benthic macroinvertebrate assemblages because they are diverse, are relatively easy to sample, and respond to changes in the physiochemical environment associated with anthropogenic disturbance (Buss et al., 2014; Hughes & Peck, 2008). To elucidate the effects of anthropogenic activities, benthic macroinvertebrate assemblages observed at a site are typically compared to reference assemblages collected from least-disturbed sites (Hawkins, 2006; Stoddard et al., 2008). Here, we develop a model-based assessment as a complementary method to evaluate how benthic macroinvertebrate assemblages may change if the effects of major anthropogenic disturbances were removed from the physiochemical environment. First, we use random forest models to relate in-stream physiochemical conditions to a suite of geoclimatic and anthropogenic factors and use these models to infer abiotic conditions if anthropogenic disturbance was eliminated. Second, we combine these estimates with JSDMs to predict how biological assemblages could change if physiochemical conditions were not altered by anthropogenic disturbance. Specifically, we evaluate the effects of changing nutrient and salinity concentrations, physical habitat, and climate on benthic macroinvertebrate assemblages because these gradients are commonly used as abiotic screens to identify least-disturbed reference sites (Herlihy et al., 2008; Mitchell et al., 2025; Paulsen et al., 2008; USEPA, 2023) and affect benthic macroinvertebrate distributions (Kopp et al., 2023).

## METHODS

For our analysis, we focused on biological and environmental data collected during the 2018/2019 National Rivers and Streams Assessment (NRSA) (Figure 1). NRSA is a collaboration between the United States Environmental Protection Agency (USEPA) and state, tribal, and federal partners to assess the chemical, physical, and biological condition of streams and rivers of the United States (https://www.epa.gov/national-aquatic-resource-surveys/nrsa). Every five years, beginning in 2008/2009, NRSA surveys ~2000 stream/river locations that are selected among nine ecoregions using a probabilistic survey design and handpicked by state and tribal partners. These sites are surveyed for biological, chemical, and physical characteristics using standardized field protocols (USEPA, 2023). Only sites included in the probabilistic sample are selected to be representative of the entire population of streams and rivers in the contiguous United States and used for assessment (Olsen & Peck, 2008). In addition, approximately 10% of the probabilistic sites are revisited to assess within-year variability, and approximately 30% of these sites are resampled in the next survey cycle to evaluate among-year variability.

### Benthic macroinvertebrate assemblages and physiochemical variables

Procedures for collecting benthic macroinvertebrates are described in detail elsewhere (Hughes & Peck, 2008; USEPA, 2017a). Briefly, each survey location was defined as a length of stream or river equal to a multiple of its channel width. In wadeable and boatable sites, the reach length sampled was equal to 40 channel widths, a minimum of 150 m or a maximum of 4 km. Macroinvertebrate samples were collected at 11 equally spaced cross-section transects along the reach. In wadeable sites, samples were collected in an alternating left, center, right order along the transects using a D-frame kick net (500-μm mesh, 0.09 m^2^ area). In boatable sites, samples were collected along the left or right wadeable margin from a 1-m linear sweep of the dominant habitat using a D-frame kick net (500-μm mesh, 0.3048 m^2^ area). Samples from each survey location were combined into a single composite sample, preserved in ethanol, and sent to a taxonomist for subsampling and identification.

To assess the effects of anthropogenic disturbance on in-stream physiochemical conditions and in turn macroinvertebrate assemblages, we focused on in situ nutrient concentrations, salinity, and physical habitat variables. These environmental gradients are commonly altered by anthropogenic activities in the surrounding watershed and frequently used as abiotic screens to identify least-disturbed reference sites (Herlihy et al., 2008; Paulsen et al., 2008; USEPA, 2023). We selected total nitrogen (NTL) and total phosphorus (PTL) as indicators of excess nutrients supplied from agriculture and/or urbanization (Herlihy et al., 1998) and chloride (CL) and sulfate (SO_4_) as indicators of salinity. Elevated CL is a general indicator of human disturbance in the catchment (Herlihy et al., 1998), and SO_4_ can indicate sites that are affected by mine drainage (Herlihy et al., 1991). Mean substrate diameter (SUBD) and riparian disturbance index (RPDI) were selected as indicators of physical habitat because human activities can increase fine sediment inputs or directly modify the riparian area (Kaufmann et al., 1999; Kaufmann, Hughes, Paulsen, Peck, Seeliger, Weber, & Mitchell, 2022). In addition, we included mean summer air temperature (Mean July and August temperature, MSAT) and total annual precipitation (TPRCP) to assess potential changes associated with differences from a 1900 to 1950 baseline. Although other factors are affected by human disturbances (e.g., metals and pesticides and hydrologic alteration), these variables provide a reasonably broad characterization of the physiochemical environment at a site (Herlihy et al., 2008; Paulsen et al., 2008).

During the survey, a single water sample was collected at each site and shipped to a central analytical laboratory. PTL and NTL were measured by persulfate digestion and colorimetry, and CL and SO_4_ were measured by ion chromatography (USEPA, 2017b). SUBD is the geometric mean of the numeric value assigned to substrate size classes measured in the field (Kaufmann et al., 1999). RPDI summarizes the presence/absence of 11 categories of human disturbance, including buildings, landfill/trash, logging, mining, developed parks or lawns, pavement or cleared lots, pipes (withdrawal or wastewater), roads, row crops, pastures or hayfields, and walls or revetments in the riparian area adjacent to each of the 11 transects where macroinvertebrates were collected (Kaufmann et al., 1999; Kaufmann, Hughes, Paulsen, Peck, Seeliger, Weber, & Mitchell, 2022; USEPA, 2017a). In addition, we included mean summer air temperature (Mean July and August temperature, MSAT) and total annual precipitation (TPRCP) for either 2018 or 2019 (depending on sample year) obtained from the PRISM Climate Group (https://prism.oregonstate.edu/).

### Modeling genus–environment relationships

We quantified relationships between macroinvertebrate assemblages and eight physiochemical variables (i.e., NTL, PTL, CL, SO_4_, SUBD, RPDI, MSAT, and TPRCP) using JSDMs fitted with the Hierarchical Modeling of Species Communities R package (Ovaskainen & Abrego, 2020; Tikhonov et al., 2020). JSDMs are multivariate hierarchical generalized linear mixed models fitted with Bayesian inference. They are uniquely suited to evaluate relationships between anthropogenic disturbance and biological assemblages because they are multi-species models that quantify taxon–environment relationships for all members in an assemblage simultaneously. They can also account for unmeasured variables, including abiotic factors and species associations, using random effects specified at the sample level (Deflem et al., 2021; Ovaskainen & Abrego, 2020; Warton et al., 2015). Indeed, sample-level random effects are meaningful for multivariate models because they are not confounded by residual variation as with univariate models. More specifically, this attribute accounts for nonindependence among residuals for each site and improves estimates of the fixed effects (i.e., taxon–environment relationships) (Ovaskainen et al., 2016). In addition, JSDMs also allow for the inclusion of phylogenetic relatedness as a hierarchical term that can lend additional improvements to estimated relationships.

We provide a detailed description of the modeling framework and application elsewhere (Kopp et al., 2023). In brief, we used presence/absence data from 1891 benthic macroinvertebrate assemblages surveyed as part of the probabilistic and handpicked sites and focused on taxa that were collected from a single site visit, identified to genus, and occurred at ≥10% of sites within an ecoregion (Table 1). Separate models were fit for each ecoregion to define separate species pools (Chessman & Royal, 2004). All physiochemical variables were measured in the field during the survey, except for MSAT and TPRCP, which were obtained from PRISM Climate and matched to the appropriate survey year (Table 1). Physiochemical variables were transformed, where necessary, to reduce skewness and used as linear fixed effects. Sample-level random effects were used to statistically control for unmeasured variables. Our analysis focused on a relatively small number of environmental variables because these are commonly altered by human activities. Previously, we found that few genus–environment relationships were unimodal (Kopp et al., 2023), and therefore, assumed linear relationships were appropriate for this study. We also use taxonomy as a surrogate for phylogenetic relatedness as a hierarchical term in the model. All models were fitted with the default prior distributions (Ovaskainen & Abrego, 2020), using three independent chains (3000 posterior samples). Convergence was determined to be satisfactory by potential scale reduction factor <1.1 for fixed effects and phylogenetic parameters.

Our primary motivation for using these models was to measure how macroinvertebrate assemblages may change if the influence of anthropogenic disturbances was removed from in-stream physiochemical variables and all else remained unchanged (Figure 2). Since we do not use these models to predict to new locations, model performance was primarily evaluated with respect to its explanatory power, that is, the fitted models ability to reproduce the observed genus richness and composition (Abrego & Ovaskainen, 2023; Wilkinson et al., 2021). Predicted, taxon-specific occurrence probabilities were summed to obtain predicted richness and regressed against the observed richness (Calabrese et al., 2014). We determined model acceptability based on the *R*^2^ and estimated intercept and slope (Linke et al., 2005). We calculated model performance metrics using the 3000 posterior samples to obtain a distribution of plausible estimates and report the mean and 5th and 95th quantiles. In addition, we assessed compositional similarity between predicted and observed assemblages using a probabilistic adaptation of Jaccard similarity (Scherrer et al., 2020) to avoid introducing error associated with converting predicted occurrence probabilities into binary outcomes (Calabrese et al., 2014). We calculated similarity for each site using the mean predicted occurrence probabilities and report the mean and 5th and 95th quantiles for all sites. We also calculated similarity indices between the predicted assemblage and using the regional genus prevalence as a null model as an additional evaluation of compositional similarity.

Predictive power for JSDM that use site-level random effects can only be assessed for cases where at least some sampling units were included in the calibration data (Abrego & Ovaskainen, 2023). As part of NRSA, a predetermined number of sites are revisited to assess temporal variability in metrics and indices (Table 1). Since we fit the JSDMs using sample-level random effects, these data were used to assess predictive power. Specifically, we evaluated whether the richness observed at a revisited site was within the posterior distribution of the fitted models. Although this metric of validation may seem less restrictive compared to those used to evaluate explanatory power, we expected variation between samples to be rather large because of stochastic events (e.g., high or low stream flows), ecological processes (e.g., emergence and dispersal), and sampling procedures (e.g., field collection and laboratory subsampling) that our models were not designed to capture. Given this level of potential uncertainty, this metric was intended to address whether an observation resulted from the same process that our model was intended to capture. We also compared the assemblages collected during the revisit to the predicted assemblages using the probabilistic adaptation of Jaccard similarity (Scherrer et al., 2020) and reported the mean and 5th and 95th quantiles of sites.

### Modeling physiochemical gradients

We used random forest models to relate anthropogenic and geoclimatic factors to NTL, PTL, CL, SO_4_, and SUBD and make predictions if anthropogenic disturbances were reduced or removed. Each response variable was transformed to reduce skewness (Table 2). The suite of predictor variables was selected based on their hypothesized relationship with stream water chemistry and bed particle size (Kaufmann, Hughes, Paulsen, Peck, Seeliger, Weber, & Mitchell, 2022; Kaushal et al., 2023; Lin et al., 2021; Sabo et al., 2023; Zak et al., 2021). Geoclimatic factors included watershed morphology (e.g., basin area, elevation, and slope) and lithological characteristics (e.g., lithological phosphate, nitrogen, sulfur, and soil erodibility). Anthropogenic factors included percent agriculture, road density, and presence of mines and impoundments (Appendix S1: Table S1). Predictor variables were obtained or derived from the StreamCat Database (Hill et al., 2016), National Atmospheric Deposition Program (nadp.slh.wisc.edu), and EPA’s National Nutrient Inventory (Lin et al., 2021; Sabo et al., 2019, 2021, 2023).

Separate random forest models were fitted to the transformed values of NTL, PTL, CL, SO_4_, or SUBD using the entire national dataset to maximize the range of the predictor variables included in each model. Prior to model fitting, the dataset was randomly split into training and testing portions (80% and 20%, respectively). Model fit was assessed by the *R*^2^ and root mean squared error (RMSE) on training and testing portions of the dataset. The relative importance of the covariates was evaluated by the change in mean squared error after permutating each variable (%IncMSE). Because variable importance can be influenced by correlated variables, we confirmed that all variables used in the models had pairwise Pearson’s correlation coefficients <0.7 and the variance inflation factors <4 (range = 2.6 and 3.9; Appendix S1: Table S3). In general, VIF > 5 indicates a potential problem with multicollinearity (O’brien, 2007). Partial dependence plots were used to visualize the relationship between the most important anthropogenic factors and each physiochemical variable. Random forest analysis was performed using the quantregForest R Package (Meinshausen, 2017).

### Evaluating effects of human disturbance on physiochemical gradients

We used the fitted random forest models to predict values for NTL, PTL, CL, SO_4_, and SUBD if anthropogenic disturbance was removed by setting all anthropogenic factors to the lowest value observed in our dataset (often zero, Appendix S1: Table S2) and leaving geoclimatic factors unchanged. Hereafter, “hindcast” refers to the reduction or removal of anthropogenic disturbance from the physiochemical variables, not necessarily a prediction to a specific historical timeframe. Although the range of predictor variables used to train the model included the values used for hindcasting, it is possible that hindcast is susceptible to extrapolation (Meyer & Pebesma, 2021; Yuan et al., 2024). To test whether the hindcast dataset was sufficiently similar to the data used to train the models, we first mean-centered and scaled all predictor variables to equivalent units (i.e., standard deviations) and weighted them according to their importance in the model. We then calculated the minimum Euclidean distance between each site in the hindcasted data and each site in the training dataset using the same center and scale (Meyer & Pebesma, 2021). The minimum value was then divided by the mean Euclidean distance among all training data. Following Yuan et al. (2024), we flagged and removed hindcast predictions for sites >0.5 as susceptible to extrapolation.

We evaluated the effects of anthropogenic disturbance on each physiochemical variable based on deviations from the hindcast estimates using standardized anomalies (i.e., z scores) or the difference between the observed and hindcast value scaled by standard deviation. For variables we modeled using random forests, we standardized the difference between observed and hindcasted conditions using the RMSE of each model (Kilgour & Stanfield, 2006). For climate variables, we obtained baseline historical climatic values (MSAT and TPRCP) as the mean summer air temperature for 1900–1950 from PRISM climate data (https://prism.oregonstate.edu/historical/). We then standardized the difference between the present-day values (i.e., 2018 or 2019) and the baseline using the standard deviation of the 50-year dataset. Importantly, using z-scores rather than absolute differences has advantages because it accounts for unexplained variation in the model or natural variability among sites (Kilgour & Stanfield, 2006). Furthermore, because the values are in units of standard deviations, evaluating an effect of anthropogenic disturbance can be rather intuitive. Specifically, we expected anthropogenic disturbance to elevate NTL, PTL, CL, and SO_4_ concentrations and increase or decrease SUBD, MSAT, and TPRCP. Accordingly, we identified sites with observed concentrations >2SD from the hindcast values for NTL, PTL, CL, and SO_4_ and >|2SD| for SUBD, MSAT, and TPRCP as having evidence of anthropogenic disturbance.

Since RPDI is a direct measure of anthropogenic disturbance, we initially set this variable to zero for all locations but found that this value may be too strict because of the number and diversity of factors included in the index and did not provide much insight into potential regional variation in disturbance. Instead, we used 0.33 as a threshold, which is interpreted as one type of human disturbance observed within 10 m of the streambanks at no more than one third of the 22 riparian plots sampled, on average (Kaufmann, Hughes, Paulsen, Peck, Seeliger, Weber, & Mitchell, 2022; USEPA, 2023). Although this does mean that not all sites were completely free of riparian disturbance, it is consistent with other studies that have used this index to evaluate human disturbance in the riparian area (Kaufmann, Hughes, Paulsen, Peck, Seeliger, Kincaid, & Mitchell, 2022; Kaufmann, Hughes, Paulsen, Peck, Seeliger, Weber, & Mitchell, 2022; USEPA, 2023).

For each physiochemical variable, we estimated the total percent of streams that were potentially affected by anthropogenic disturbance (i.e., >2SD) using only the probabilistic samples and their weights that reflect the entire population of streams and rivers (USEPA, 2023). Estimates for each ecoregion were generated using the cat_analysis() function from the spsurvey R package (Dumelle et al., 2023). In addition, we tallied the number of physiochemical variables that were potentially affected at each site to elucidate instances where human disturbance affects multiple environmental variables simultaneously.

### Evaluating effects of human disturbance on macroinvertebrate assemblages

We evaluated the effects of removing anthropogenic disturbance from the physiochemical environment on macroinvertebrate assemblages using the fitted JSDMs. For the sites that had a z-score >2SD, we substituted the hindcasted value (either predicted from random forest model, 1900–1950 averages for MSAT and TPRCP or 0.33 for RPDI) for the present-day value in the dataset and used these data to predict macroinvertebrate assemblages that could occur if disturbance was removed or reduced (Chessman & Royal, 2004). To evaluate the relative effects of hindcasting each physiochemical variable, we created four scenarios changing either NTL and PTL (nutrients), CL and SO_4_ (salinity), RPDI and SUBD (habitat), and MSAT and TPRCP (climate) and leaving the others at their observed values. In addition, we predicted the macroinvertebrate assemblage after changing all physiochemical variables to their hindcast value. For each scenario, we compared predicted genus richness from present-day conditions to hindcast genus richness.

The JSDM predictions are three-dimensional data arrays that contain 3000 posterior samples of site-specific occurrence probabilities for each genus (i.e., site × genera × posterior samples). For each posterior sample, we summed predicted occurrence probabilities for all genera to obtain a posterior distribution of hindcast genus richness for each site. We then compared the mean present-day genus richness (i.e., predicted from JSDM using present-day values for the physiochemical variables) to the hindcast posterior distribution. We evaluated whether present-day richness was below the 10th or 25th quantile, indicating a reduction in present-day genus richness relative to hindcast predictions, or above the 75th or 90th quantile, indicating an increase in genus richness relative to hindcasted (Figure 2). Hereafter, present-day values that are located in the extreme ends of the distribution are described as having either >0.75 or >0.90 support for a difference from hindcasted predictions, respectively.

Site-specific occurrence probabilities for each genus help interpret assemblage-level changes that richness alone might not capture. We identified genera that had higher occurrence probabilities under present-day conditions as “increasers” and genera with lower occurrence probabilities as “decreasers.” Differences were determined with >90% support because we expected greater uncertainty in individual occurrence probabilities compared to assemblage-level richness metrics. For each genus, we calculated the proportion of sites where they increased or decreased. If a genus was identified as an increaser at a large proportion of sites, it could become more prevalent in response to anthropogenic disturbance. Alternatively, if a genus was identified as a decreaser at a large proportion of sites, it could become less prevalent in response to human disturbances. For each ecoregion, we report the mean for major taxonomic groups (e.g., insects and noninsect genera) to further understand taxon-specific trends (Jähnig et al., 2021; Rumschlag et al., 2023).

Increaser and decreaser genera also enhance our ability to identify changes in assemblage composition without a subsequent change in richness (Van Sickle, 2008). To assess compositional differences between present-day and hindcast assemblages, we used two community matrices populated with increaser/decreaser assignments. Increasers were assigned a value of 1 in the “present-day” matrix and 0 in the “hindcast” matrix because they had a higher occurrence probability under present-day conditions. Conversely, decreasers were assigned 0 in the “present-day” matrix and 1 in the “hindcast” matrix because they had a significantly higher occurrence probability under hindcasted conditions. Genera that did not have sufficient support for changing occurrence probabilities were assigned 1 in both matrices because they were considered not to be affected by anthropogenic disturbance. We compared the two matrices using the Jaccard similarity index and identified sites with a similarity score of <0.9 as changing compositionally.

We assessed the consequences of removing anthropogenic disturbance from the in-stream physiochemical environment for macroinvertebrate assemblages by identifying sites within each region that had evidence for either a change in richness (>0.75 support) or composition (Jaccard Similarity <0.9) from present-day conditions. Then, using the NRSA survey design weights for samples that were not flagged for extrapolation and had complete data (n = 1748), we estimated the total percent of streams within each ecoregion that were potentially affected by anthropogenic disturbance. Sites where the extrapolation potential was high or had incomplete data were designated as “Not Assessed.” Estimates for each ecoregion were generated using the cat_analysis() function from the spsurvey R package (Dumelle et al., 2023).

## RESULTS

### Random forest modeling

The random forest models explained 46%–77% of the variation in the training data (n = 1502) and 51%–78% of the variation in the test/validation data (n = 375). The model of SUBD had the highest RMSE in both training and testing datasets compared to models of NTL, PTL, CL, and SO_4_ (Table 2). Variable importance for each physiochemical model, measured by the percent change in MSE after permutation, revealed that runoff was among the most important geoclimatic variables for each model and that agricultural land cover in the watershed or riparian area ranked among the most important anthropogenic variables (Appendix S1: Table S2). Percent agricultural land cover in the watershed was the most important variable predicting NTL and PTL, the second most important for predicting SO_4_, and third most important for predicting CL. Other anthropogenic variables were also ranked relatively high in importance. For example, road density in the watershed was the second most important variable predicting CL and the fifth most important variable for SO_4_, while percent natural vegetation cover in the riparian area was the third most important variable predicting SUBD.

We visualized the effects of the anthropogenic factors using partial dependence plots (Figure 3). In general, the anthropogenic factors were associated with increased nutrients and salinity and decreased SUBD. For example, percent crops and anthropogenic nutrient inputs of nitrogen and phosphorus were positively associated with NTL and PTL. Road density and mean summer air temperature had a positive association with CL, while total annual precipitation had a weakly negative relationship. The density of coal mines in the watershed had a relatively strong positive effect on SO_4_, and SUBD had a strong positive relationship with natural vegetation cover and a strong negative relationship with agricultural activities in the riparian area.

### JSDM assemblage-level performance

We fitted JSDMs for each region using presence/absence data for 59–127 genera with >10% prevalence (20%–30% of entire regional taxa pool) collected from 152 to 266 sites surveyed (Table 1). Typically, rare taxa that were excluded from the analysis accounted for <26% of the assemblage collected at a site (average proportion: CPL, 0.25; NAP, 0.20; NPL, 0.23; SAP, 0.19; SPL, 0.26; TPL, 0.22; UMW, 0.20; WMT, 0.25; XER, 0.26). Sites that had assemblages consisting of only regionally rare taxa were not used for modeling. Nonetheless, the total number of sites used to fit JSDMs was higher (1891) than the sites we ultimately assessed because we included probabilistic and handpicked sites (USEPA, 2023).

We found that summing predicted occurrence probabilities for all genera modeled could potentially overestimate observed genus richness (i.e., observed vs. predicted richness intercepts <−1.5). Upon further inspection, we found that this overprediction was due to many genera having exceptionally low predicted occurrence probabilities but nonetheless present in the modeled species pool. Thus, we used a threshold to exclude genera with predicted occurrence probabilities <0.05 or <0.10 prior to summation to correct this bias (Table 3).

Because site-level random effects accounted for unmeasured factors in the models, we did not expect our models to have strong predictive power when projected to new locations (Abrego & Ovaskainen, 2023; Kopp et al., 2023). However, we could assess whether values obtained at revisited sites were within the predicted posterior distribution to complement our analysis of explanatory power. Although the number of revisited sites available for model testing was low (Table 1), we found that >90% of the genus richness values observed during revisit sampling were within the posterior distribution of the models. Importantly, this lends plausibility that the posterior distribution reflects processes characterizing macroinvertebrate assemblages (Table 3; Appendix S1: Figure S2).

The probabilistic Jaccard similarity revealed that the predicted and observed assemblages were more similar (>50%) to the observed assemblage compared to the null expectation (Table 3). Sites located in the WMT tended to have the most similar assemblages (mean = 0.6, 5^th^ quantile = 0.36, 95th quantile = 0.75), while sites in the XER tended to have the least similarity (mean = 0.23, 5th quantile = 0.23, 95th quantile = 0.71). Although potentially vulnerable to sample size constraints, mean similarity between model predictions and assemblages observed at revisited sites was also higher than the null expectation (Table 3).

### Disturbance effects on physiochemical conditions

We tested whether using the random forest models for hindcasting physiochemical conditions was susceptible to extrapolation. We found that predictions at 77 of the sites (~4%) were potentially susceptible to extrapolation. This indicates that removing disturbance would cause predictions for some sites to be extrapolations. Indeed, most of the sites flagged for potential extrapolation were located in the Coastal Plains Ecoregions (18%) and were associated with models used to predict NTL concentrations in the lower Mississippi and SUBD along the southern coast (Appendix S1: Figure S1). To avoid the potential for bias associated with extrapolation, we designated them as “Not Assessed” in our analysis.

For sites that were not potentially susceptible to extrapolation, removing anthropogenic disturbance from the physiochemical environment generally decreased nutrient concentrations, salinity, and riparian disturbance and increased SUBD (Figure 4). Change in NTL and PTL concentrations was most evident in Temperate Plains and Southern Plains, and change in CL was most evident in the Northern Appalachians. SUBD was noticeably coarser after hindcasting for all regions except Xeric, Southern Appalachians, and Northern Appalachians. Observed and hindcasted values for sulfate and mean summer air temperature were generally similar for all ecoregions.

Anthropogenic disturbance affected physiochemical gradients differently depending on the ecoregion (Figure 5A). We found that 60.8% (95% CI = 52.8%–68.8%) of streams in the Temperate Plains and 38.4% (CI = 32.0%–44.9%) of streams in the Upper Midwest had elevated NTL relative to hindcasted estimates. In the Northern Appalachians, 41.4% (95% CI = 34.5%–48.2%) of streams were found to have potentially elevated CL concentrations. Streams with excess fine substrates were most prevalent in the Temperate Plains (29.2%, 95% CI = 21.2%–37.1%) and Northern Plains (21.4%, 95% CI = 14.1%–28.9%). We also found similarities among regions. RPDI and TPRCP were the most prevalent disturbances among all ecoregions, while SO_4_ and MSAT were least prevalent. Anthropogenic disturbance could alter multiple physiochemical variables simultaneously (Figure 5B). In general, sites with >2 physiochemical variables disturbed by anthropogenic activities were located in the Northern Appalachians, Southern Plains, Temperate Plains, and Upper Midwest.

### Macroinvertebrate assemblage response to undisturbed physiochemical environment

Macroinvertebrate models were fit for each region separately, and we assessed whether removing anthropogenic disturbance would produce estimates that exceed the range of values used to fit each model. Hindcast predictions exceeded the range of present-day conditions at 1 location in CPL, SAP, and XER, 6 locations in TPL, and 12 locations in WMT. In these instances, we reset the hindcasted values to be within the range (minimum) of data used to fit the model to avoid potential extrapolation.

Hindcast predictions for each category of physiochemical variables generated different outcomes for macroinvertebrate assemblages (Figure 6A). For example, removing anthropogenic disturbance from NTL and PTL concentrations produced macroinvertebrate assemblages with higher genus richness in the Temperate Plains and lower richness at some sites in the Southern Plains and Southern Appalachians ecoregions (Figure 6A). On the other hand, removing disturbance from CL and SO_4_ tended to increase genus richness in the Southern Plains but decreased richness at sites in the Northern Appalachians. Hindcasted physical habitat variables mostly affected macroinvertebrate assemblages in the Northern Plains by decreasing richness but also increased genus richness in the Coastal Plains, Northern Appalachians, and Upper Midwest (Figure 6A). TPRCP generally contributed to changes in macroinvertebrate assemblages because relatively few sites had MSAT temperature anomalies exceed 2 standard deviations from the 1900–1950 mean. In the Southern Appalachians, genus richness decreased from present-day conditions, while in the Northern Plains, genus richness generally increased from present-day conditions. Removing hindcasting all physiochemical variables typically increased genus richness in all regions except for the Northern Plains and Southern Appalachians (Figure 6B).

Identifying genera that had higher present-day occurrence probabilities (increasers) or higher hindcast occurrence probabilities (decreasers) indicated that insects tended to be decreasers while noninsects tended to be increasers, but this was not consistent across all regions (Table 4). For example, in the Southern Plains and Western Mountains, insects were almost equally likely to be increasers (0.1 and 0.6, respectively) or decreasers (0.09 and 0.03, respectively), and in the Northern Plains, both insects and noninsects tended to be increasers at a larger proportion of sites (0.19 and 0.22). Among insects, members of Chironomidae were generally most likely to be increasers. Among noninsects, members of Mollusca were most likely increasers (Table 4). Thus, higher present-day genus richness compared to hindcast estimates could be due to genera that are typically considered to be tolerant of human activities.

Using the probabilistic sites, we aggregated our results to reflect the total population of streams and rivers in each ecoregion and detected that 14.3%–75.5% of streams have present-day assemblages that differ from assemblages expected from hindcasting (Figure 7). With respect to changes in richness, the Northern Plains (58.3%), Temperate Plains (54.8%), and Southern Plains (54.2%) had the most streams with potentially altered macroinvertebrate assemblages, while the Western Mountains (9.2%), Upper Midwest (19.6%), and Northern Appalachians (29.7%) had the fewest. Compositional change without a corresponding change in genus richness could increase the percentage of streams with altered assemblages. Evidence for compositional change was most pronounced in the Temperate Plains (20.7%), Southern Appalachians (20.7%), and Upper Midwest (18.8%). All regions had a percentage of streams that could not be assessed because of potential extrapolation, incomplete data, or macroinvertebrate assemblages consisting of only rare genera (i.e., prevalence <10%). Among them, the Coastal Plains (12.9%) and Xeric (11.1%) had the largest percentage of streams that were not assessed; thus, the total number of sites assessed was less than the total number of probabilistic sites surveyed (n = 1748).

## DISCUSSION

Assessing the effects of anthropogenic disturbance on biological assemblages is challenging because few minimally disturbed sites remain (Stoddard et al., 2006). Sites in least-disturbed condition are often adopted as an alternative but are difficult to consistently define, vary in quality, and can be spatially aggregated (Hawkins et al., 2010; McNellie et al., 2020). The model-based framework we developed contributes to a suite of approaches intended to circumvent the need for least-disturbed reference sites (Chessman & Royal, 2004; Elias et al., 2016; Yuan et al., 2024). However, because there is a paucity of historical and/or experimental data on physiochemical and biological conditions in the absence of human disturbance (Hawkins et al., 2010), they are extremely difficult to validate using traditional methods (Vander Laan & Hawkins, 2014). Nonetheless, models fitted to present-day conditions can be a useful heuristic in space-for-time substitution contexts (Blüthgen et al., 2022). Here, we inferred the effects of human disturbance on several physiochemical variables and, in turn, addressed whether altered physiochemical conditions affect macroinvertebrate assemblages. Using this approach, we found that anthropogenic disturbance can affect multiple physiochemical variables simultaneously and that the effects on any single factor can vary among ecoregions. We also found that removing or reducing disturbance could change genus richness at >50% of the streams in some ecoregions and up to 75% if compositional change is considered. Collectively, our framework offers a promising, albeit exploratory, framework to evaluate the effects of specific disturbances on macroinvertebrate assemblages without relying on least-disturbed reference sites.

### Hindcasting physiochemical conditions

Random forest models have been used by others to predict physiochemical variables if human disturbance was reduced or removed (Yuan et al., 2024). In general, using models to infer physiochemical conditions eliminates the need to identify reference sites or collect excessive data from undisturbed locations (Herlihy & Sifneos, 2008; Soranno et al., 2011). In this study, random forest models improve on earlier regression-based hindcasting approaches (Dodds & Oakes, 2004; Herlihy & Sifneos, 2008) because they incorporate a relatively large number of natural and anthropogenic predictor variables and accommodate their complex relationships with the response variables (Yuan et al., 2024). Importantly, because reference sites may not be representative of all the sites that need to be assessed, including natural and anthropogenic variables enabled us to make site-specific predictions rather than an estimated mean value for an entire region (Dodds & Oakes, 2004, Herlihy & Sifneos, 2008). Thus, withstanding concerns regarding potential extrapolation issues associated with random forest models (Booker & Whitehead, 2018; Hateffard et al., 2024; Meyer & Pebesma, 2021), our hindcasting approach provides a first-order approximation for what conditions at a site could be if they are presently unknown.

Furthermore, random forest models permitted us to evaluate the relative importance of specific anthropogenic activities and characterize potential site and regional scale differences after reducing or removing them. For example, due to higher than expected NTL levels in Temperate Plains and Upper Midwest and the relatively high importance of percent agriculture land cover, our model suggests that nutrient inputs associated with agricultural activities may be elevating in-stream nutrient concentrations (Lin et al., 2021; Sabo et al., 2023). Similarly, elevated chloride concentrations in the Northern Appalachians and the relative high importance of road density in the model could reflect the contribution of road salts to freshwater salinization (Kaushal et al., 2023).

Certainly, inferring physiochemical conditions for different levels of human disturbance depends on the quality and structure of the model. Although our models were evaluated using an independent validation dataset, hindcasting required a dataset that reduced or eliminated the effect of predictor variables associated with human disturbance. As a consequence, the dataset used for hindcasting could be dissimilar from the data used to calibrate the model and potentially susceptible to extrapolation (Booker & Whitehead, 2018, Hateffard et al., 2024, Meyer & Pebesma, 2021). Tree-based models, including random forests, generally underperform when extrapolating to novel environmental space, but there is no readily available method for assessing whether predictions are within or beyond the training data (Booker & Whitehead, 2018). Using a relatively straightforward approach that has been used in similar studies (Yuan et al., 2024), we found that for most regions, the hindcasting dataset was sufficiently similar to the training dataset such that our predictions were closer to interpolation. Nonetheless, 18% of sites surveyed in the Coastal Plains were flagged as a result of testing for extrapolation. Considering that many of the sites were located in the lower Mississippi River Basin, a unique and heavily modified system, it is perhaps unsurprising that removing human disturbances from this system is beyond the domain of our model. Importantly, testing for potential extrapolation in the context of hindcasting is novel, and more research should be devoted to optimizing and interpreting this approach and threshold to more confidently assess extrapolation. When locations or streams are potentially susceptible to extrapolation, it is more reasonable to select physiochemical values that represent management goals, best professional judgment, or maximize societal benefits (Bouleau & Pont, 2015).

To assess how human-related disturbance affects the physiochemical environment, we used standardized anomalies (i.e., z-scores) to account for either unexplained variation in the model values or variation along a baseline. Thresholds are of central importance for communication and evaluation of human disturbances (Wood, 2008). Here, we used a ±2 SD threshold for deciphering whether human disturbances alter physiochemical conditions (Kilgour & Stanfield, 2006). Although this threshold is intuitive, it could potentially be too conservative for some variables. For example, values for MSAT at many locations did not exceed the threshold in our analysis, suggesting that 2018 and 2019 temperature averages are consistent with 1900–1950 averages, given interannual variation. However, warming of 1 SD has been implicated in reductions of insects in agricultural landscapes (Outhwaite et al., 2022), and there is evidence for increasing summer air temperatures in the United States of approximately 0.09°C per decade since 1901 (USEPA, 2024a). On the other hand, to evaluate riparian disturbance, we used a threshold of 0.33, which suggests that human disturbance is not completely absent for all sites that were below this threshold. Indeed, nearly every site in our analysis has RPDI > 0, such that this threshold did not informatively differentiate regional variation in disturbance. Further, this is the threshold used by others to identify least-disturbed sites (Kaufmann, Hughes, Paulsen, Peck, Seeliger, Weber, & Mitchell, 2022; USEPA, 2023), and we sought to evaluate how this could affect microinvertebrate assemblages. Although thresholds were necessary to identify which regions may have relatively more disturbed physiochemical conditions than others, future applications may select different thresholds, report continuous scores, or vary the amount of disturbance to meet specific applications (Yuan et al., 2024).

### Application for biological assessment without reference sites

Using hindcast physiochemical variables to assess how assemblages might change if anthropogenic disturbance was removed allowed us to infer the consequences of altering a relatively small number of physiochemical variables. Central to our application is that multivariate models can implement meaningful site-level random effects that statistically control for unmeasured environmental variables and potential biotic interactions (Kopp et al., 2023; Ovaskainen et al., 2016; Warton et al., 2015). Although this feature kept our model sufficiently tractable, it limits our ability to make accurate predictions to new locations (Abrego & Ovaskainen, 2023). However, predicting to new locations was not our objective. Rather, our models establish empirical relationships between macroinvertebrate occurrences and then evaluate how those assemblages might differ if select physiochemical conditions changed and all else remained constant. Furthermore, because the data we used for our analysis were collected as a part of a probabilistic survey (Olsen & Peck, 2008), our site-specific inferences can be aggregated to elucidate regional and sub-continental patterns. Indeed, it may be undesirable to refit models every time an assessment is needed, and future efforts should focus on improving predictive abilities by including immutable factors in addition to stressor gradients (Yuan et al., 2024).

Importantly, our approach differs significantly from traditional, reference site-based approaches (Hawkins et al., 2000; Herlihy et al., 2008; USEPA, 2023). Foremost, our efforts focus explicitly on a relatively small number of potential stressors, whereas reference-site approaches focus implicitly on a theoretically larger but undefined number of stressors. For example, the difference between a test site and reference sites could be related to a number of other stressors that were not used as biotic screens but co-occur with them (Herlihy et al., 2008; Paulsen et al., 2008). Alternatively, in our model-based approach, differences between present day and hindcast assemblages are only related to changes in the physiochemical variables included as fixed effects. Although the former may elucidate general disturbance effects, it is difficult to attribute a specific environmental disturbance (Paulsen et al., 2008). On the other hand, our model-based approach enhances interpretations with respect to specific physiochemical variables but may omit important anthropogenic stressors that were not explicitly included in the model. Thus, the choice between reference site-based and model-based assessments may be contingent on whether the environmental gradients that are commonly disturbed by human activities can be identified and appropriately modeled.

The JSDMs were also fitted using Bayesian inference and, as such, yielded posterior distributions that were used to evaluate whether the effects of human disturbance on physiochemical variables were sufficient to alter benthic assemblages for each site. By contrast, reference site-based approaches evaluate each test site based on quantiles of the distribution obtained from reference sites (Herlihy et al., 2008, USEPA, 2023). In this regard, JSDMs and Bayesian inference may be advantageous for interpreting biological condition estimates because they explicitly account for prediction uncertainty at each site. Furthermore, we included all genera from the regional taxa pool that had a prevalence >10%, while typical reference site-based approaches include only taxa that occur at reference sites. This implies that in the absence of human disturbance, reference taxa should occur at all locations. This is potentially problematic because genera that tolerate anthropogenic disturbance probably evolved under similar conditions that occurred naturally or have remarkable plasticity (Heino et al., 2013; Wiens et al., 2010). Finally, our model-based approach produces genus-specific occurrence probabilities that can reveal compositional changes. Although we found that predicting individual genera is more challenging than aggregated genus richness, this analysis provides additional information that is generally not readily available from other approaches (Hawkins, 2006; Van Sickle, 2008).

Selecting the appropriate taxonomic level for biological condition assessments is a critical decision (Chessman et al., 2007). We focused exclusively on the genus level and excluded taxa that could not be unambiguously identified. This could increase false absence rates in our study, but NRSA identifies most organisms to their genus such that these instances are relatively rare. Further, using taxa that were unambiguously identified alleviates some ambiguity associated with aggregating unresolved taxa into operational taxonomic units (Yuan et al., 2008) and improves transferability of our genus–environment relationships to other studies. Fixed-count subsampling, performed as part of the standardized NRSA protocol, could also increase false absence in our study. Although excluding rare taxa may resolve this problem to some extent, explicitly modeling taxon-specific detection probabilities from replicate subsamples may be an interesting avenue for future research (Doser et al., 2023, 2024). Unfortunately, these data are presently unavailable. Finally, focusing on genera could also mask interspecific variation. In general, finer taxonomic resolution would be substantially more expensive and perhaps only yield marginal benefits (Chessman et al., 2007). Nonetheless, metabarcoding approaches have shown promise for bioassessments (Smucker et al., 2024) and could avoid some of the limitations associated with selecting the appropriate taxonomic resolution.

Although the model-based approaches may have some advantages over reference site-based approaches, they require further investigation before they can be assimilated into biomonitoring programs. This includes using models that may be better at extrapolation for hindcasting physiochemical conditions and adding immutable factors to increase the predictive power of JSDM. Nevertheless, the National Rivers and Streams Assessment reports biological condition estimates for the 2018–2019 survey using a multi-metric index (USEPA, 2024b), and it is worthwhile to compare our results to those. The results from NRSA are available at https://riverstreamassessment.epa.gov/dashboard. Based on a ranked comparison, the largest disagreements pertained to the percentage of stream miles in poor condition in the Plains ecoregions. Specifically, our model-based approach found more streams to be altered in the Northern Plains, Southern Plains, and Temperate Plains. Indeed, the US Great Plains (i.e., Temperate Plains, Northern Plains, and Southern Plains) have undergone extensive conversion from grasslands to agriculture such that there may be few sites that are undisturbed (Dodds et al., 2004; Olimb & Robinson, 2019; Samson & Knopf, 1994). Because of the large extent of anthropogenic activities in these regions, it is likely that the reference sites used to assess biological condition are potentially lower quality (Herlihy et al., 2008). Conversely, NRSA ranked the Coastal Plains as having a larger percentage of streams in poor condition. Unfortunately, the Coastal Plains also had a relatively large number of streams that we did not assess because they were flagged for extrapolation. The rankings for the remaining ecoregions tended to be similar, and both methods agreed that streams in the Western Mountains were the least disturbed among the ecoregions. Indeed, Western Mountains have a relatively shorter history of anthropogenic disturbance than other ecoregions in the United States and about 75% of the land area is in federal ownership, which could convey some protection to streams and rivers (Jenkins et al., 2015).

### Macroinvertebrate response to anthropogenic disturbance

Beyond applications for bioassessment, our model-based approach contributes to a growing literature devoted to understanding recent trends in macroinvertebrates (Crossley et al., 2020; Gebert et al., 2022; Jähnig et al., 2021; Rumschlag et al., 2023; Spake et al., 2022). In our study, hindcasting estimates tended to show increases in genus richness, suggesting that in many ecoregions fewer genera persist under present-day conditions. Other studies have reported either modest increases in macroinvertebrate richness (Gebert et al., 2022; Rumschlag et al., 2023) or found no clear evidence for a widespread decline of insects (Crossley et al., 2020). Because few datasets span more than several decades, an advantage of space for time substitutions is that they could reflect conditions before major disturbances (Blüthgen et al., 2022). In addition, the survey design implemented by NRSA reduces the potential for confounding spatial and temporal dimensions that could be present in datasets that were compiled from multiple sources (Blüthgen et al., 2022; Boyd et al., 2023; Jähnig et al., 2021). Nonetheless, although our results suggest that sites typically support fewer macroinvertebrate genera under present-day conditions, we did detect increases in genus richness at several sites in the Northern Plains and Western Mountains, which suggests that macroinvertebrate response to disturbance could be context dependent (Powell et al., 2023).

We compared genus-specific occurrence probabilities under present-day and hindcasted conditions to identify which genera contribute to assemblage-level patterns. Insects in most ecoregions tended to decrease with disturbance, while noninsects tended to increase. This suggests that insect genera may, in general, be less tolerant to anthropogenic disturbance (Jähnig et al., 2021) and aligns with other studies that have documented declines in insect richness (Rumschlag et al., 2023). Among insects, Ephemeroptera, Plecoptera, and Trichoptera (EPT) are often used as indicators of anthropogenic disturbance (Stoddard et al., 2008) and may have already lost a considerable proportion of species (Sânchez-Bayo & Wyckhuys, 2019). This is especially concerning because we found that EPT occurrence probabilities could continue to decrease in many regions of the United States. Among noninsects, members of Annelida, Nemertea, and Platyhelminthes tended to increase at a relatively large proportion of sites, which is consistent with high pollution tolerance values typically assigned to these taxa (Carlisle et al., 2007; Griffith, 2023).

Because our approach was based on quantifying genus–environment relationships along environmental gradients that are typically altered by human activity, we could also compare the relative effects of changing specific categories of physiochemical variables on macroinvertebrate assemblages. For example, higher genus richness under present-day conditions in the Northern Plains compared to hindcasted habitat variables suggests that disturbance may have increased genera richness. Interestingly, cattle in pasture and rangelands were among the most common types of disturbance measured by RPDI for this region (USEPA, 2023) and could potentially increase genera richness by suppressing riparian forest cover and, in turn, elevating primary production (Mittelbach et al., 2001; Tonkin et al., 2013). Conversely, in the Northern Appalachians and Coastal Plains, where the presence of trash, landfills, or buildings was the most common factor for RPDI, we found that genus richness was presently lower, which is consistent with the negative effects of urbanization on stream macroinvertebrate assemblages (Morse et al., 2003). We also found different effects with respect to changes in precipitation whereby genus richness increased in the Northern Plains and decreased in the Southern Appalachians. Both regions have slightly higher present-day precipitation levels, but the Northern Plains is more arid. If organisms in this region are adapted to arid conditions, higher precipitation could be considered a stressor and reduce macroinvertebrate richness. Alternatively, in the Southern Appalachians, organisms may already be adapted to wetter conditions such that more precipitation creates more habitat and increases species richness. Collectively, these patterns reveal the relative importance of environmental variables for structuring microinvertebrate assemblages and in what contexts—interpretations that would not be possible with traditional reference site-based approaches.

## CONCLUSIONS

We used a model-based approach to assess the potential effects of anthropogenic disturbance on physiochemical gradients and benthic macroinvertebrate assemblages. The number of sites in minimally disturbed condition is progressively diminishing, and least-disturbed reference sites can be difficult to identify, spatially biased, and variable in quality. Novel methods and concepts are urgently needed to overcome these challenges and more fully understand the extent of anthropogenic disturbances and track the status and trends of ecological systems. Model-based assessments could provide an avenue forward but would benefit from future research.

## Supplementary Material

Supplement1

Additional supporting information can be found online in the Supporting Information section at the end of this article.

## Figures and Tables

**FIGURE 1:**
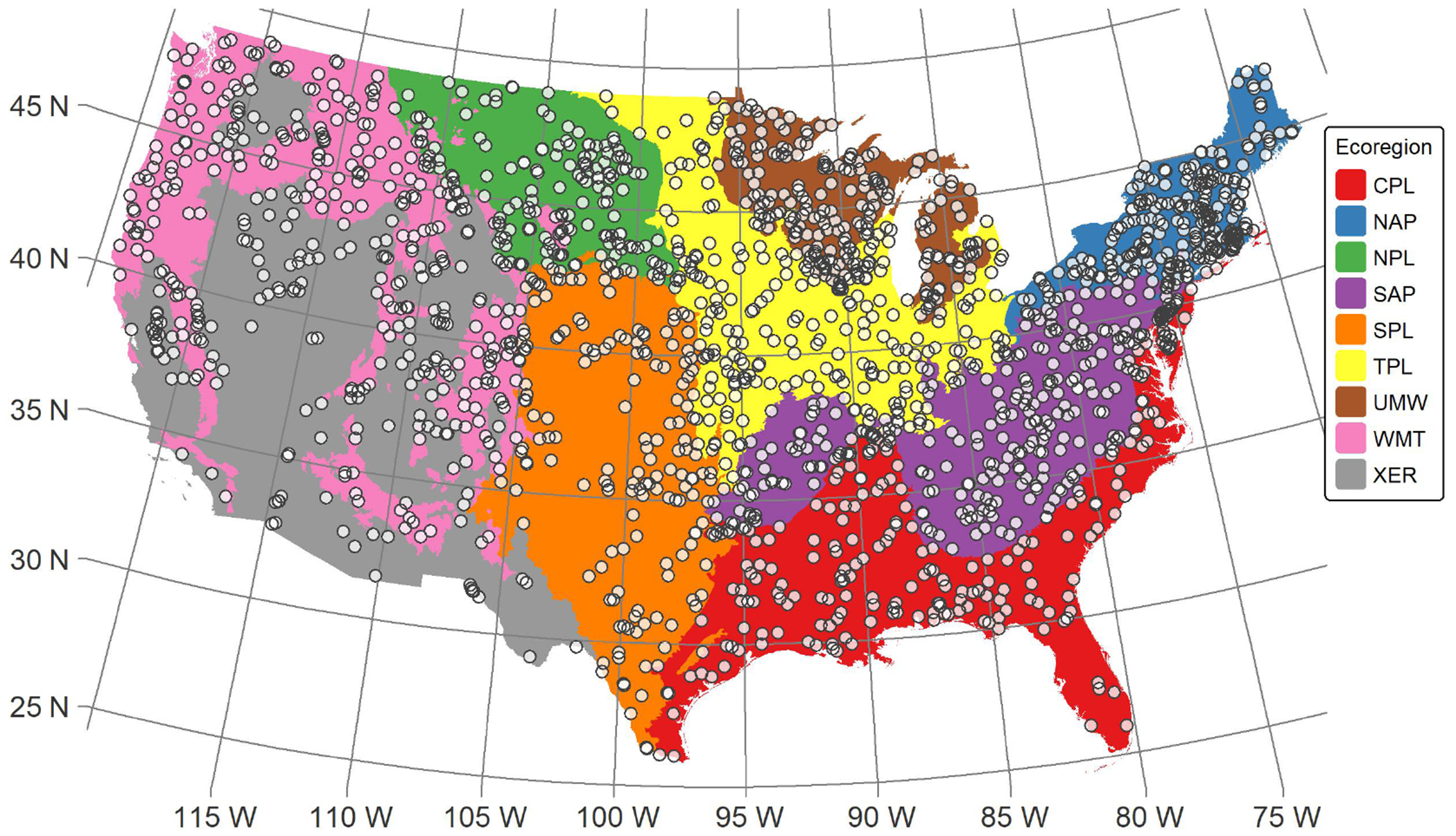
Ecoregions and survey locations for the 2018–2019 National Rivers and Streams Assessment. CPL, Coastal Plains; NAP, Northern Appalachians; NPL, Northern Plains; SAP, Southern Appalachians; SPL, Southern Plains; TPL, Temperate Plains; UMW, Upper Midwest; WMT, Western Mountains; XER, Xeric.

**FIGURE 2:**
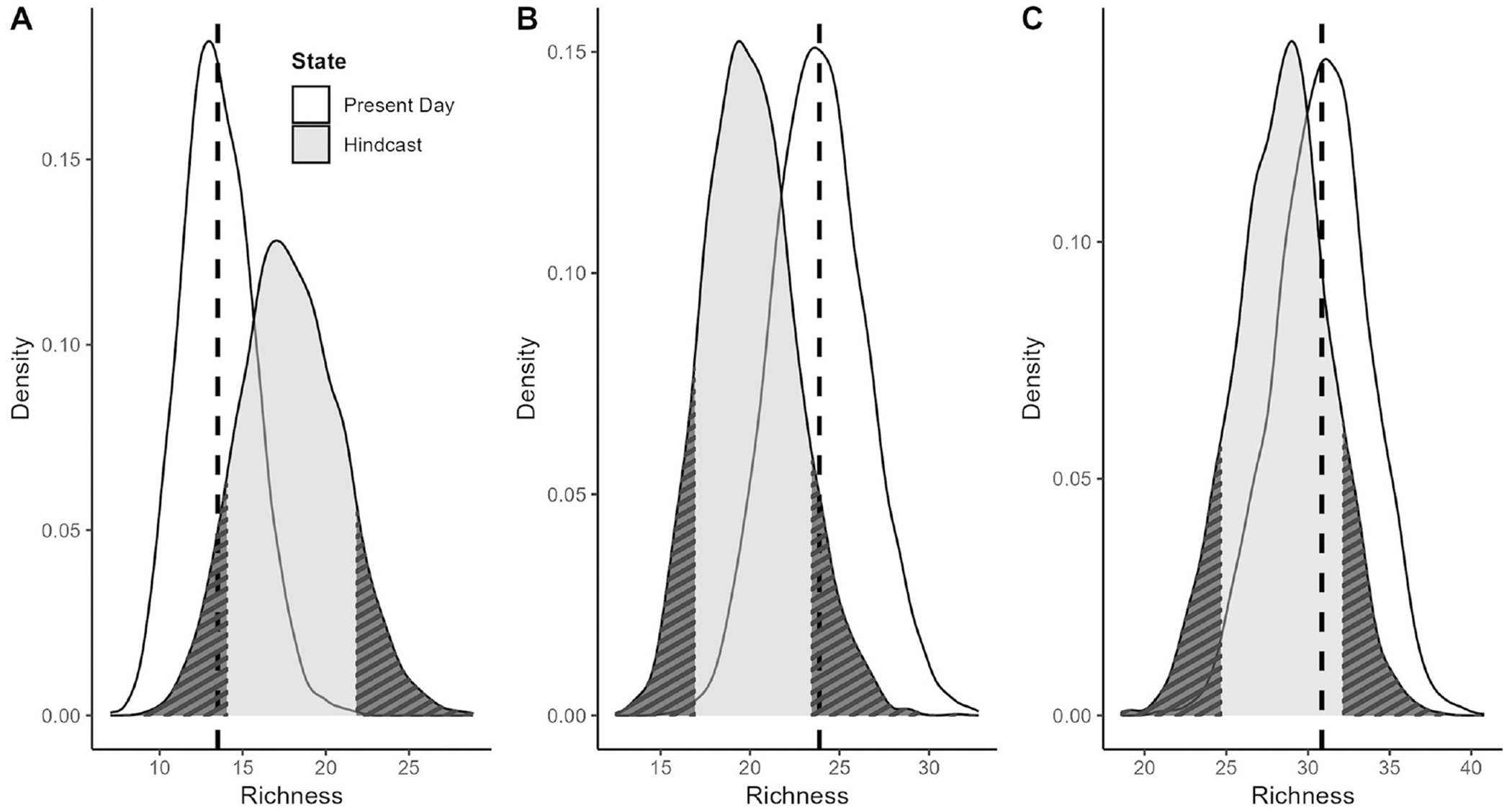
A conceptual diagram of posterior distributions used for assessment at three sites. Gray is a hindcast distribution of genus richness generated after removing anthropogenic disturbances, and white is a posterior distribution generated from present-day conditions. The dotted line is the mean of the present-day distribution, and the dark shading represents the lower 10th and upper 90th quantiles of the hindcast distribution. Site (A) has a present-day mean <10th quantile of the hindcast distribution, which indicates that present-day richness is lower than hindcasted richness. Alternatively, site (B) has richness >90th quantile of the hindcast distribution and indicates that present-day richness is likely higher. At site (C), the two distributions are similar, suggesting that there is likely no difference between the present-day and hindcast assemblages.

**FIGURE 3:**
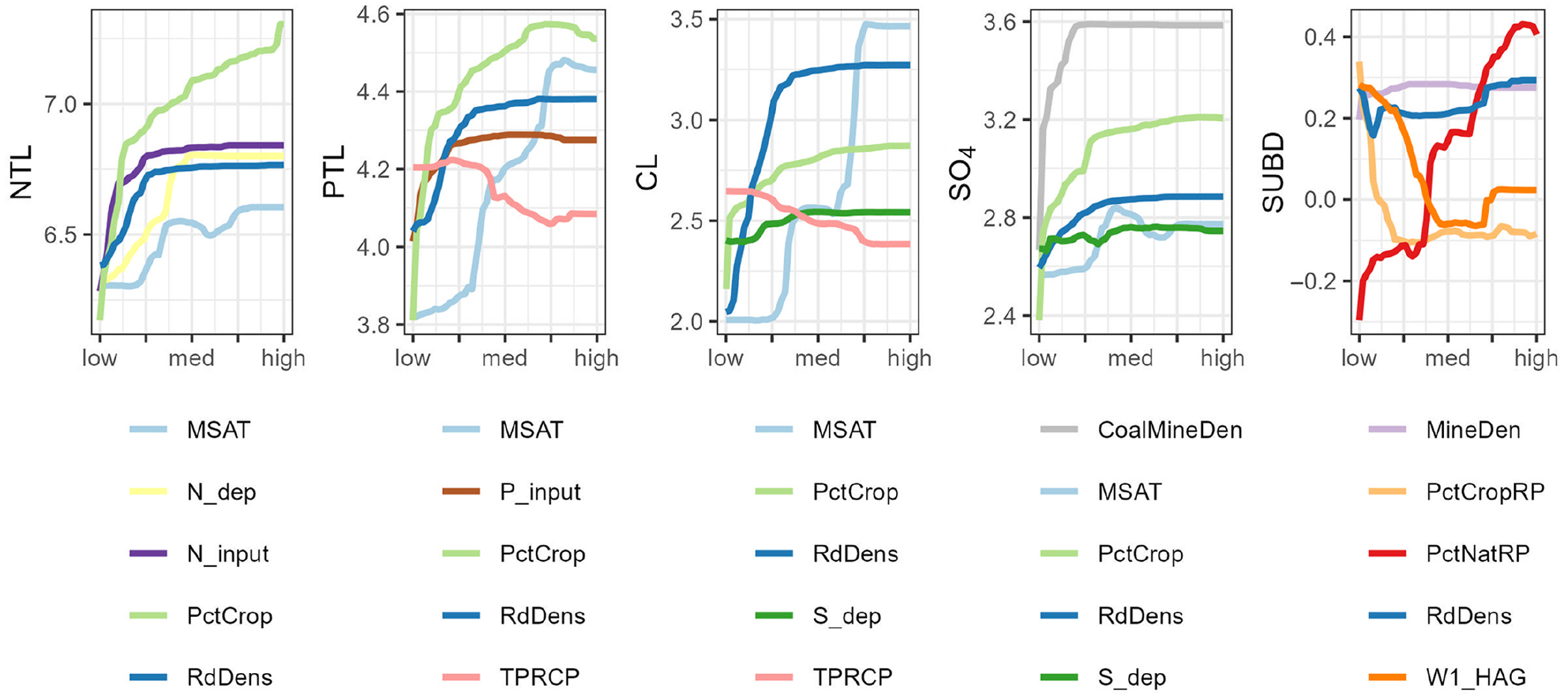
Partial dependence plots showing the effects of anthropogenic variables on in-stream physiochemical factors. For visualization, each anthropogenic factor was rescaled between 0 and 1 and labeled low, medium, and high. CL, chloride (in milligrams per liter); CoalMineDen, coal mine density; MineDen, gravel mine density; MSAT, mean summer air temperature; N_dep, atmospheric nitrogen deposition; N_input, anthropogenic nitrogen inputs; NTL, total nitrogen (in micrograms per liter); P_input, anthropogenic phosphorus inputs; PctCrop, percent crop in the watershed; PctCropRP, percent crop in the riparian area; PctNatRP, percent natural vegetation in the riparian area; PTL, total phosphorus (in micrograms per liter); RdDen, road density; S_dep, atmospheric sulfur deposition; SO_4_, sulfate (in milligrams per liter); SUBD, substrate diameter log(millimeters); TPRCP, total precipitation; W1_HAG, agricultural disturbance adjacent to the stream reach.

**FIGURE 4:**
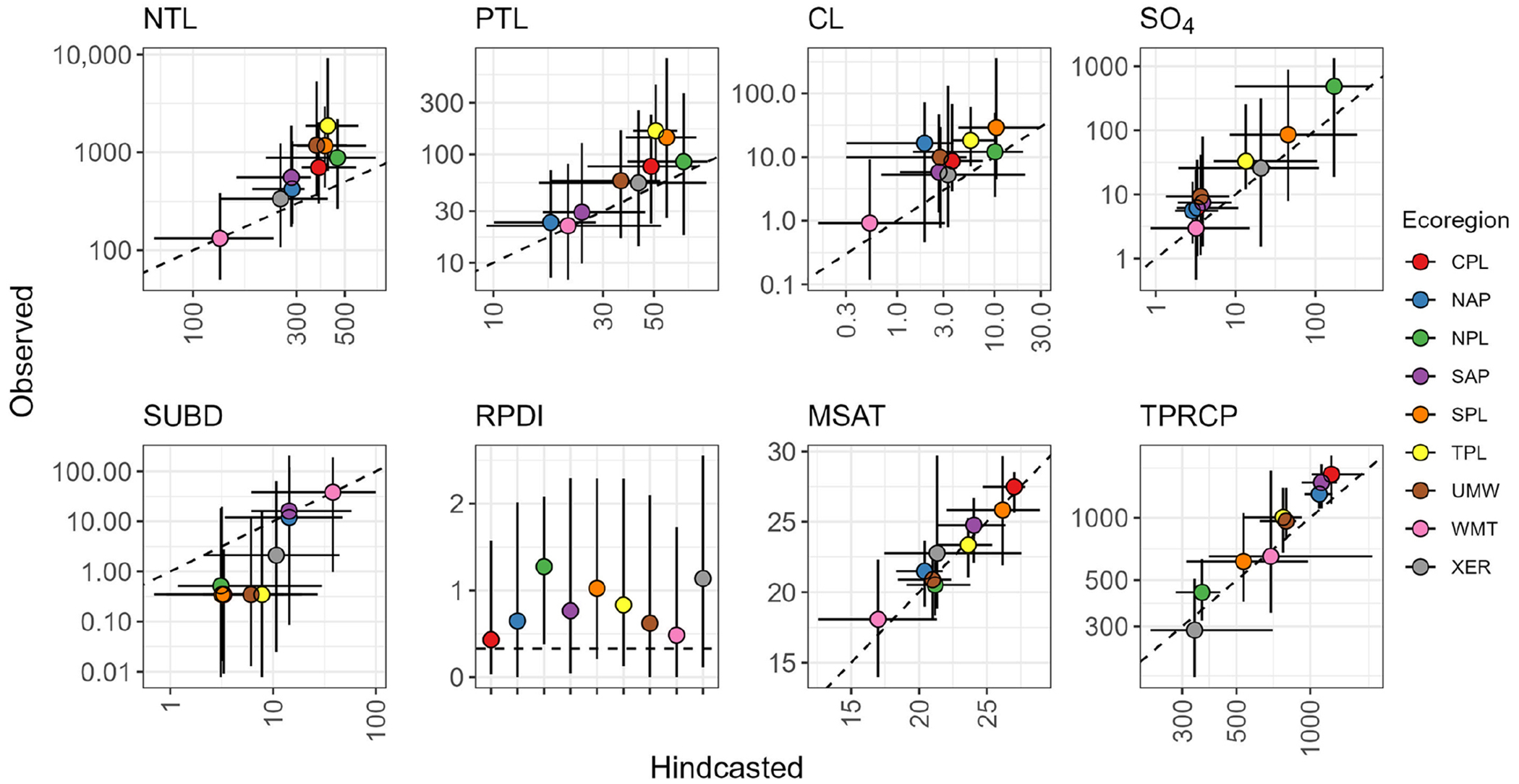
Observed versus hindcasted values for each environmental gradient. Points are regional means, and vertical and horizontal bars represent the 10th and 90th quantiles of observed or hindcasted values, respectively. For riparian disturbance index (RPDI), all hindcast values were <0.33, and the ecoregions were plotted separately for visualization. The dashed line is the 1:1 relationship for all plots except for RPDI, where it represents the 0.33 threshold applied to all ecoregions. CL, chloride (in milligrams per liter); CPL, Coastal Plains; MSAT, mean summer air temperature (in degrees Celsius); NAP, Northern Appalachians; NPL, Northern Plains; NTL, total nitrogen (in micrograms per liter); PTL, total phosphorus (in micrograms per liter); SAP, Southern Appalachians; SO_4_, sulfate (in milligrams per liter); SPL, Southern Plains; SUBD, substrate diameter (in millimeters); TPL, Temperate Plains; TPRCP, total precipitation (mm); UMW, Upper Midwest; WMT, Western Mountains; XER, Xeric. See Appendix S1: Table S4 for observed and hindcasted mean values and quantiles for each region.

**FIGURE 5:**
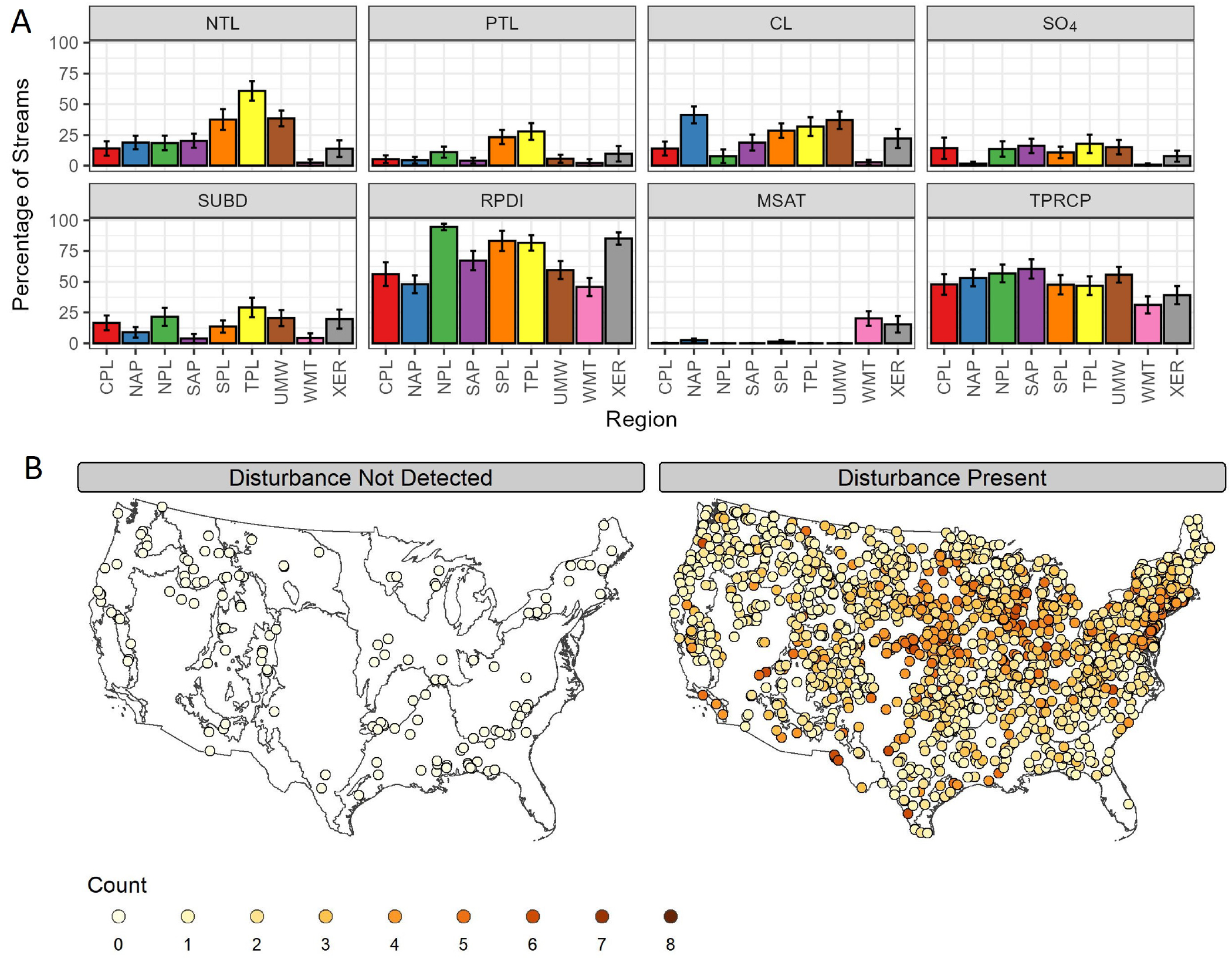
(A) Proportion of sites where hindcasted abiotic conditions were >2 SD from the observed value for total nitrogen (NTL), total phosphorus (PTL), chloride (CL), sulfate (SO_4_), substrate diameter (SUBD), mean summer air temperature (MSAT), and total precipitation (TPRCP) and >0.33 for riparian disturbance index (RPDI). (B) The number of environmental variables affected by human disturbance. CPL, Coastal Plains; NAP, Northern Appalachians; NPL, Northern Plains; SAP, Southern Appalachians; SPL, Southern Plains; TPL, Temperate Plains; UMW, Upper Midwest; WMT, Western Mountains; XER, Xeric.

**FIGURE 6:**
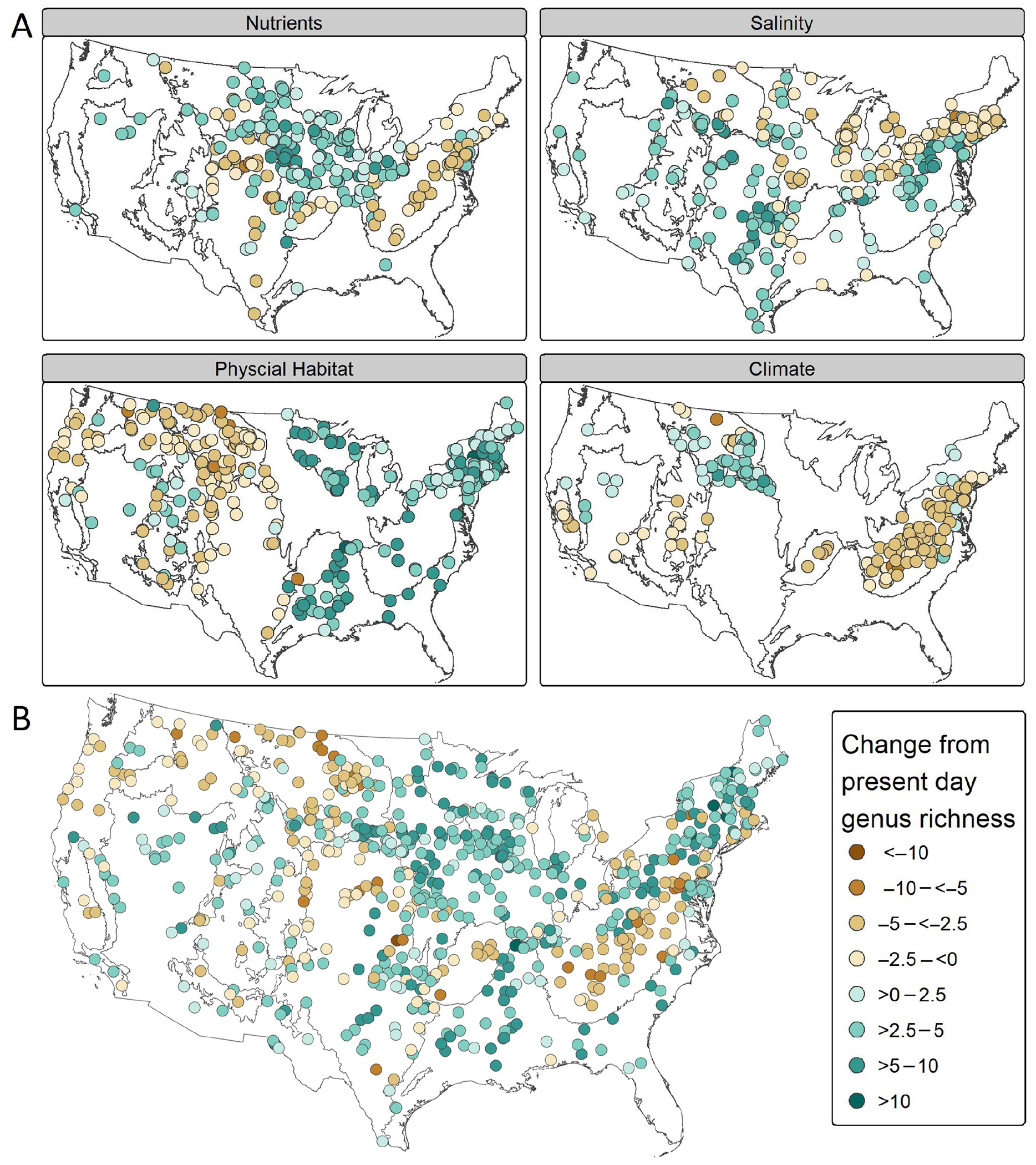
Difference in macroinvertebrate genus richness after removing anthropogenic disturbance from (A) each category of physiochemical variables and (B) all variables simultaneously. The points in each panel are sites that changed in genus richness with >75% support.

**FIGURE 7:**
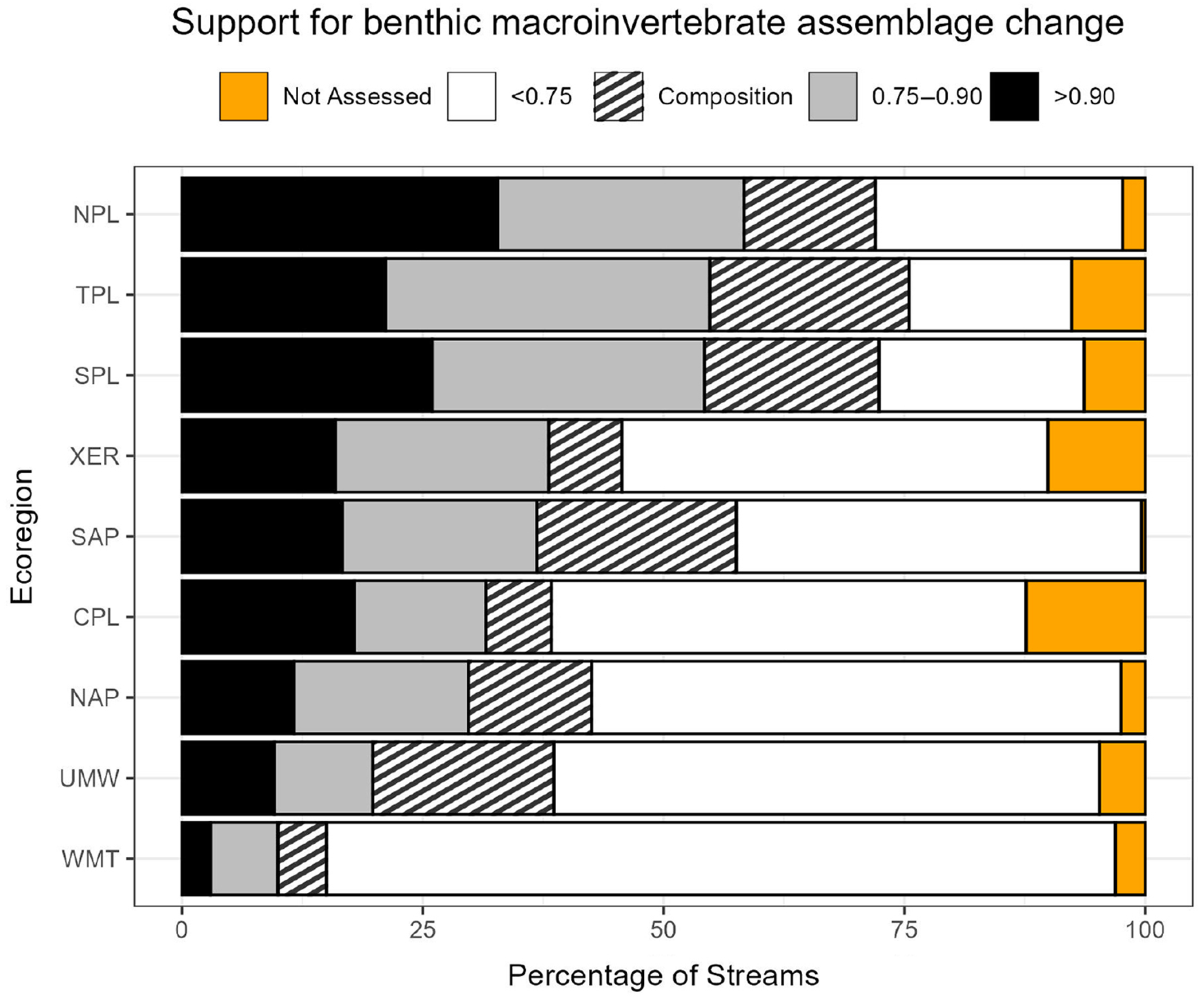
Percentage of streams where genus richness or composition could change from present-day. Shaded bars indicate that the probability of mean present-day richness differs from hindcast with >0.90 (black) or 0.75–0.90 (gray) support. Hatched bars indicate that Jaccard similarity was <0.9 but support for a difference in richness were <0.75. White bars indicate the proportion of sites that had <0.75 support for change in richness and >0.9 compositional similarity. Orange bars indicate the proportion of sites that could not be assessed because of insufficient data or potential extrapolation from predicting hindcast physiochemical conditions. CPL, Coastal Plains; NAP, Northern Appalachians; NPL, Northern Plains; SAP, Southern Appalachians; SPL, Southern Plains; TPL, Temperate Plains; UMW, Upper Midwest; WMT, Western Mountains; XER, Xeric.

**TABLE 1:** Summary of variables used for joint species distribution modeling.

Region	No. sites	No. genera	NTL (μg/L)	PTL (μg/L)	CL (mg/L)	SO_4_ (mg/L)	RPDI	SUBD (mm)	TPRCP (mm)	MSAT (°C)
CPL	226 (36)	71 (22)	786 (91–7713)	84.81 (5.2–3922.25)	10.06 (0.52–4797.9)	7.83 (0.11–1900.89)	0.5 (0–5.39)	0.35 (0.01–341.65)	1613.83 (388.66–2397.1)	27.46 (23.75–31.68)
NAP	228 (27)	127 (32)	431 (81–6413)	23.76 (3.12–587.2)	16.73 (0.07–668.16)	5.58 (0.06–467.93)	0.65 (0–5.24)	11.66 (0.01–864.9)	1295.41 (888.91–1883.17)	21.49 (17.24–24.72)
NPL	152 (7)	76 (26)	881.5 (71–15,675)	85.61 (3.53–9248.31)	12.09 (0.11–1251.58)	486.24 (3.5–4079.78)	1.27 (0–4.49)	0.51 (0.01–560.5)	437.42 (157.84–1055.11)	20.52 (15.62–23.46)
SAP	266 (32)	111 (30)	557.5 (36–18,700)	29.3 (3.79–4050)	5.89 (0.36–197.39)	7.46 (0.58–397.69)	0.76 (0–4.56)	16.28 (0.01–5656.85)	1475.78 (889.64–2451.81)	24.76 (19.19–27.73)
SPL	174 (5)	59 (20)	1165 (145–21,175)	147.12 (5.21–4351.7)	30.36 (0.43–5220)	88.89 (1.96–3716.6)	1.04 (0–5.88)	0.35 (0.01–5656.85)	616.17 (240.1–1404.61)	25.88 (11.96–32.75)
TPL	223 (29)	74 (25)	1806 (236–16,219)	165.08 (11.45–1066.87)	18.9 (1.44–736.62)	34.4 (5.79–1386.77)	0.83 (0–5.47)	0.35 (0.01–5656.85)	1004.22 (376.7–1801.08)	23.33 (18.28–27.09)
UMW	201 (10)	104 (28)	1168 (195–17,675)	57.4 (8.16–659)	9.97 (0.01–306.69)	9.28 (0.04–160.4)	0.62 (0–6.43)	0.35 (0.01–812.79)	960.16 (0.01–812.79)	20.84 (17.05–23.43)
WMT	225 (19)	94 (24)	133 (22–4719)	21.84 (2.71–569.8)	0.92 (0.04–521.64)	2.97 (0.07–1682.05)	0.48 (0–3.95)	38.14 (0.01–1288.61)	669.31 (175.89–3946.33)	18.08 (11.71–29.4)
XER	196 (16)	75 (21)	344.5 (46–8000)	54.62 (4.29–4667.41)	5.35 (0.1–1867.57)	27.8 (0.02–3286.24)	1.16 (0–4.36)	2.02 (0.01–368.11)	287.58(100.46–1176.56)	22.76 (14.69–36.11)

*Note*: Values for sites and genera are counts, and environmental variables are medians for each region. The number of revisit sites refers to locations that were revisited during the survey used for validation, given as counts in parenthesis. The number of genera with >10% prevalence is a percentage of the regional taxa given in parenthesis. The range for each environmental variable is given in parentheses.

Abbreviations: CL, chloride; CPL, Coastal Plains; MSAT, mean summer air temperature; NAP, Northern Appalachians; NPL, Northern Plains; NTL, total nitrogen; PTL, total phosphorus; RPDI, riparian disturbance index; SAP, Southern Appalachians; SO_4_, sulfate; SPL, Southern Plains; SUBD, substrate diameter; TPL, Temperate Plains; TPRCP, total precipitation; UMW, Upper Midwest; WMT, Western Mountains; XER, Xeric.

**TABLE 2:** Random forest model performance metrics for testing and out-of-bag training datasets.

Variable	*R* ^2^_train_	RMSE_train_	*R* ^2^_test_	RMSE_test_
ln(NTL + 1) (μg/L)	0.73	0.61	0.72	0.60
ln(PTL + 1) (μg/L)	0.60	0.78	0.69	0.70
ln(CL + 1) (mg/L)	0.73	0.75	0.68	0.85
ln(SO_4_) (mg/L)	0.77	0.96	0.78	0.97
log_10_(SUBD) (mm)	0.46	0.98	0.51	1.00

Abbreviations: CL, chloride; NTL, total nitrogen; PTL, total phosphorus; RMSE, root mean squared error of random forest models fitted with ln(*x* + 1) (NTL, PTL, and CL) or ln(*x*) (SO_4_) or log_10_(SUBD) transformations; SO_4_, sulfate; SUBD, substrate diameter.

**TABLE 3:** Mean regression coefficients for predicted and observed richness and mean compositional similarity.

Region	Pr	Genus richness				Jaccard similarity		
		Intercept	Slope	*R* ^2^	Revisit sites^a^	Null	All sites	Revisit sites
CPL	0.05	0.37 (−0.37–1.02)	1.00 (0.96–1.05)	0.85 (0.82–0.87)	97	0.38 (0.13–0.58)	0.52 (0.26–0.7)	0.68 (0.24–0.95)
NAP	0.05	−0.45 (−2.02–1.02)	1.03 (0.99–1.08)	0.74 (0.71–0.78)	90	0.35 (0.18–0.51)	0.52 (0.31–0.64)	0.35 (0.17–0.65)
NPL	0.05	−0.07 (−1.21–1.04)	1.02 (0.96–1.09)	0.76 (0.71–0.81)	100	0.36 (0.16–0.55)	0.58 (0.34–0.77)	0.39 (0.12–0.71)
SAP	0.05	0.40 (−0.66–1.41)	1.00 (0.97–1.04)	0.85 (0.82–0.87)	97	0.38 (0.16–0.58)	0.52 (0.26–0.67)	0.56 (0.21–0.87)
SPL	0.10	0.48 (−0.45–1.29)	1.02 (0.97–1.09)	0.79 (0.75–0.83)	100	0.36 (0.1–0.58)	0.52 (0.25–0.72)	0.61 (0.52–0.98)
TPL	0.05	0.06 (−0.91–0.94)	1.01 (0.97–1.06)	0.82 (0.79–0.84)	100	0.39 (0.16–0.63)	0.55 (0.31–0.77)	0.59 (0.18–1)
UMW	0.05	−0.74 (−2.52–0.99)	1.04 (0.98–1.10)	0.73 (0.66–0.78)	100	0.4 (0.21–0.56)	0.55 (0.34–0.69)	0.53 (0.27–0.9)
WMT	0.10	0.24 (−1.24–1.59)	1.04 (0.99–1.10)	0.74 (0.70–0.78)	100	0.41 (0.15–0.57)	0.60 (0.36–0.75)	0.45 (0.16–0.79)
XER	0.05	−0.12 (−1.11–0.74)	1.03 (0.98–1.08)	0.83 (0.80–0.86)	94	0.32 (0.08–0.53)	0.50 (0.23–0.71)	0.48 (0.07–0.83)

*Note*: Occurrence probability thresholds (Pr) were used to exclude rare genera. For genus richness, values in parentheses are the 5th and 95th percentiles of the posterior distribution. For composition, values in parentheses are the 5th and 95th percentiles of values measured for all sites. Null refers to similarity calculated using regional prevalence; all sites refer to similarity between observed and modeled assemblages; and revisit sites refer to assemblages collected during a revisit.

a Within posterior distribution (%).

**TABLE 4:** Mean proportion of sites with increaser or decreaser genera by major taxonomic group.

Genera	*D*	*I*
CPL		
Insects	0.16	0.08
EPT	0.24	0.01
Chironomidae	0.11	0.09
Other insects	0.21	0.07
Noninsects	0.14	0.16
Arthropoda	0.21	0.11
Mollusca	0.08	0.11
Other noninsects	0.15	0.22

NAP		
Insects	0.08	0.05
EPT	0.07	0.04
Chironomidae	0.09	0.05
Other insects	0.08	0.08
Noninsects	0.06	0.18
Arthropoda	0.07	0.16
Mollusca	0.03	0.28
Other noninsects	…	0.14

NPL		
Insects	0.1	0.17
EPT	0.17	0.11
Chironomidae	0.04	0.18
Other insects	0.07	0.21
Noninsects	0.06	0.19
Arthropoda	0.11	0.02
Mollusca	0.02	0.3
Other noninsects	0.05	0.13

SAP		
Insects	0.1	0.11
EPT	0.13	0.08
Chironomidae	0.04	0.12
Other insects	0.13	0.12
Noninsects	0.11	0.13
Arthropoda	0.16	0.09
Mollusca	0.1	0.15
Other noninsects	0.04	0.16

SPL		
Insects	0.09	0.12
EPT	0.09	0.04
Chironomidae	0.02	0.17
Other insects	0.23	0.02
Noninsects	0.06	0.13
Arthropoda	0.05	0.06
Mollusca	0.09	0.06
Other noninsects	0.02	0.19

TPL		
Insects	0.16	0.11
EPT	0.23	0.04
Chironomidae	0.12	0.14
Other insects	0.18	0.11
Noninsects	0.15	0.15
Arthropoda	0.23	0.35
Mollusca	0.21	0.05
Other noninsects	0.05	0.15

UMW		
Insects	0.11	0.12
EPT	0.14	0.03
Chironomidae	0.06	0.16
Other insects	0.09	0.04
Noninsects	0.08	0.14
Arthropoda	0.1	0.04
Mollusca	0.14	0.07
Other noninsects	0.01	0.25

WMT		
Insects	0.03	0.06
EPT	0.04	0.06
Chironomidae	0.02	0.07
Other insects	0.02	0.03
Noninsects	0.03	0.08
Arthropoda	0.04	0.03
Mollusca	0.01	0.06
Other noninsects	0.04	0.14

XER		
Insects	0.09	0.05
EPT	0.1	0.03
Chironomidae	0.06	0.1
Other insects	0.11	0.01
Noninsects	0.04	0.14
Arthropoda	0.05	0.04
Mollusca	0.01	0.26
Other noninsects	0.04	0.14

*Note*: Increasers (I) are genera that have a higher probability of occurrence under present-day conditions compared to hindcasted conditions; decreasers (D) are genera that have a lower probability of occurrence under present-day conditions compared to hindcasted conditions.

Abbreviations: CPL, Coastal Plains; EPT, Ephemeroptera; Plecoptera, and Trichoptera; NAP, Northern Appalachians; NPL, Northern Plains; SAP, Southern Appalachians; SPL, Southern Plains; TPL, Temperate Plains; UMW, Upper Midwest; WMT, Western Mountains; XER, xeric.

## Data Availability

Benthic macroinvertebrate occurrence data and physiochemical variables were retrieved from the 2018–2019 National Rivers and Stream Assessment (NARS): https://www.epa.gov/national-aquatic-resource-surveys/data-national-aquatic-resource-surveys. Landscape variables were retrieved from the U.S. Environmental Protection Agency NARS: https://www.epa.gov/national-aquatic-resource-surveys/streamcat-metrics-and-definitions and PRISM Climate Group: https://prism.oregonstate.edu/.
