# Supplementary material for: A model-based assessment of anthropogenic disturbance on lotic macroinvertebrate assemblages
Source: Ecosphere. Author manuscript; Available in PMC 2026 Oct 5. (PMC12973236; doi:10.1002/ecs2.70361)
Supplement: Supplementary file 1 [file NIHMS2127611-supplement-Supplement1.pdf]

Ecosphere

A model-based assessment of anthropogenic disturbance on lotic macroinvertebrate assemblages

Darin A. Kopp, John L. Stoddard, Philip R. Kaufmann, Alan T. Herlihy, Ryan A. Hill, Meredith M. Brehob, Robert D. Sabo

Appendix S1

Table S1: Geoclimatic and anthropogenic variables used in random forest models. Variables were selected based on their hypothesized relationship with total nitrogen, total phosphorus, chloride, sulfate and substrate diameter and were obtained or derived from the StreamCat Database (Hill et al. 2016), National Atmospheric Deposition Program (nadp.slh.wisc.edu), EPA's National Nutrient Inventory (Sabo et al. 2019, Sabo et al. 2021, Sabo et al. 2023) and field data collected with the National Rivers and Streams Assessment.

Variable	Description	Source
BFIWs	The component of streamflow that can be attributed to ground-water discharge	StreamCat
ClayWs	Mean % clay content of soils in watershed	StreamCat
ElevWs	Mean elevation of watershed (m)	StreamCat
KffactWs	Mean of STATSGO Kffactor	StreamCat
DepClWs	Mean atmospheric chloride deposition 2018	National Atmospheric Deposition Program
DepPWs	Atmospheric phosphorous deposition 2013	National Nutrient Inventory
NWs	Rock derived nitrogen in watershed	StreamCat
P2O5Ws	Mean % of lithological phosphorous oxide (P2O5) content in surface or near surface geology	StreamCat
PctAlluvCoastWs	% of watershed area classified with lithology type as alluvium and fine-textured coastal zone sediment	StreamCat
PctHbWetWs	% of watershed area classified as herbaceous wetland land cover	StreamCat
PermWs	Mean permeability (cm/hour) of soils (STATSGO) within watershed	StreamCat
RunoffWs	Mean runoff (mm) within watershed	StreamCat
SandWs	Mean % sand content of soils (STATSGO) within watershed	StreamCat
StreamPower	An index reflecting the amount of energy the water exerts on the sides and bottom of a stream	(((TPRCP - ET)/1000) *(0.032*WSArea)^0.5) * slope
SWs	Mean % of lithological sulfur (S) content in surface or near surface geology within watershed	StreamCat
WsAreaSqKm	Area of watershed (square km)	StreamCat
CoalMineDensWs	Density of coal mines sites within watershed (mines/square km)	StreamCat
DepNWs	Mean atmospheric nitrogen deposition in watershed 2018	National Atmospheric Deposition Program
DepSWs	Mean atmospheric sulfur deposition in watershed 2018	National Atmospheric Deposition Program
MineDensWs	Density of coal mines sites within watershed (mines/square km)	StreamCat

N_inputs	Sum of anthropogenic nitrogen inputs: N_Fert_FarmWs, N_Fert_UrbanWs, N_Human_WasteWs, N_Livestock_WasteWs	National Nutrient Inventory
P_inputs	Sum of anthropogenic phosphorus inputs: P_f_fertilizerWs, P_human_wasteWs, P_livestock_WasteWs, P_nf_fertilizerWs	National Nutrient Inventory
PctCropWs	% of watershed area classified as crop land use	StreamCat
PctCropWsRp100	% of watershed riparian area classified as crop land use	StreamCat
PctNatWs	% of watershed with natural vegetation cover	StreamCat
TPRCP	Ln(Mean total annual precipitation, 2018 or 2019)	PRISM
RdDensWs	Density of roads (2010 Census Tiger Lines) within watershed (km/square km)	StreamCat
MSAT	Mean summer (July/August) air temperature (C°), 2018 or 2019	PRISM
W1_HAG	Agricultural disturbance adjacent to a stream	NRSA Field Data
NABD_NrmStorWs	Volume all reservoirs per unit area of watershed (cubic meters/square km)	StreamCat

Table S2: Variable importance rankings for random forest models and values used to estimate physiochemical conditions in the absence of anthropogenic disturbance. The 5 most important variables for each model are bold.

Predictor Variable	NTL	PTL	CL	SO4	SUBD	Range of Predictor Present-day	Value Without Disturbance
**Geoclimatic**							
BFIWs	6	13	7	8	-	2.6 - 88	--
ClayWs	-	10	8	12	-	2.35 - 60.6	--
ElevWs	10	**4**	6	9	-	2.02 - 3587.98	--
KffactWs	13	11	15	14	9	0.01 - 0.48	--
DepClWs	-	-	9	-	-	0.13-43.16	--
DepPWs	-	**5**	-	-	-	2.03-15.9	--
NWs	9	-	11	**4**	-	0-1.59	--
P2O5Ws	16	9	18	15	-	0.02-2.42	--
PctAlluvCoastWs	-	-	-	-	6	0-100	--
PctHbWetWs	7	8	10	16	-	0-40.35	--
PermWs	14	14	17	13	-	0.32-45.23	--

RunoffWs	**2**	**2**	**4**	**1**	**2**	0.05-3731.74	--
SandWs	-	-	-	-	8	2.8-92.04	--
StreamPower	-	-	-	-	**1**	0-0.44	--
SWs	12	15	**5**	**3**	-	0.01-23.66	--
WsAreaSqKm	15	12	14	11	**5**	0.21-2,874,021.46	--
**Anthropogenic**							
CoalMineDensWs	-	-	19	7	13	0 - 5.74	0
DepNWs	**5**	-	-	-	-	1.12 - 26.15	5 kgN/ha/yr*
DepSWs	-	-	12	10	-	0.22 - 5.88	1.65 kgS/ha/yr*
MineDensWs	-	-	16	18	12	0 - 0.13	0
N_inputs	**3**	-	-	-	-	0 - 6.22	0
P_inputs	-	7	-	-	-	0 - 2.19	0
PctCropWs	**1**	**1**	**3**	**2**	-	0 - 96.7	0
PctCropWsRp100	-	-	-	-	**4**	0 - 95.52	0
PctNatWs	-	-	-	-	**3**	0 - 100	100
TPRCP	11	16	13	17	-	4.61 - 8.28	Average 1900-1950
RdDensWs	**4**	6	**2**	5	10	0 - 15.15	0
MSAT	8	**3**	**1**	6	-	11.71 - 36.11	Average 1900-1950
W1_HAG	-	-	-	-	7	0 - 2.18	0
NABD_NrmStorWs	-	-	-	-	11	0 – 1,886,390.68	0

* Estimates for atmospheric nitrogen and sulfur deposition were obtained from (Clark et al. 2018). Although they used 0.4 kgN/ha and 0.1kgS/ha no sites had deposition values below this level. Instead, we selected the higher values of 3-5KgN/ha as reasonable estimates for background deposition values before 1900. For S deposition, other efforts have estimated pre-industrial S deposition at 0.32–2.98 kgS/ha (Granat et al. 1976, Fakhraei et al. 2016) and we used the used middle number of this interval as a reasonable estimate for background.

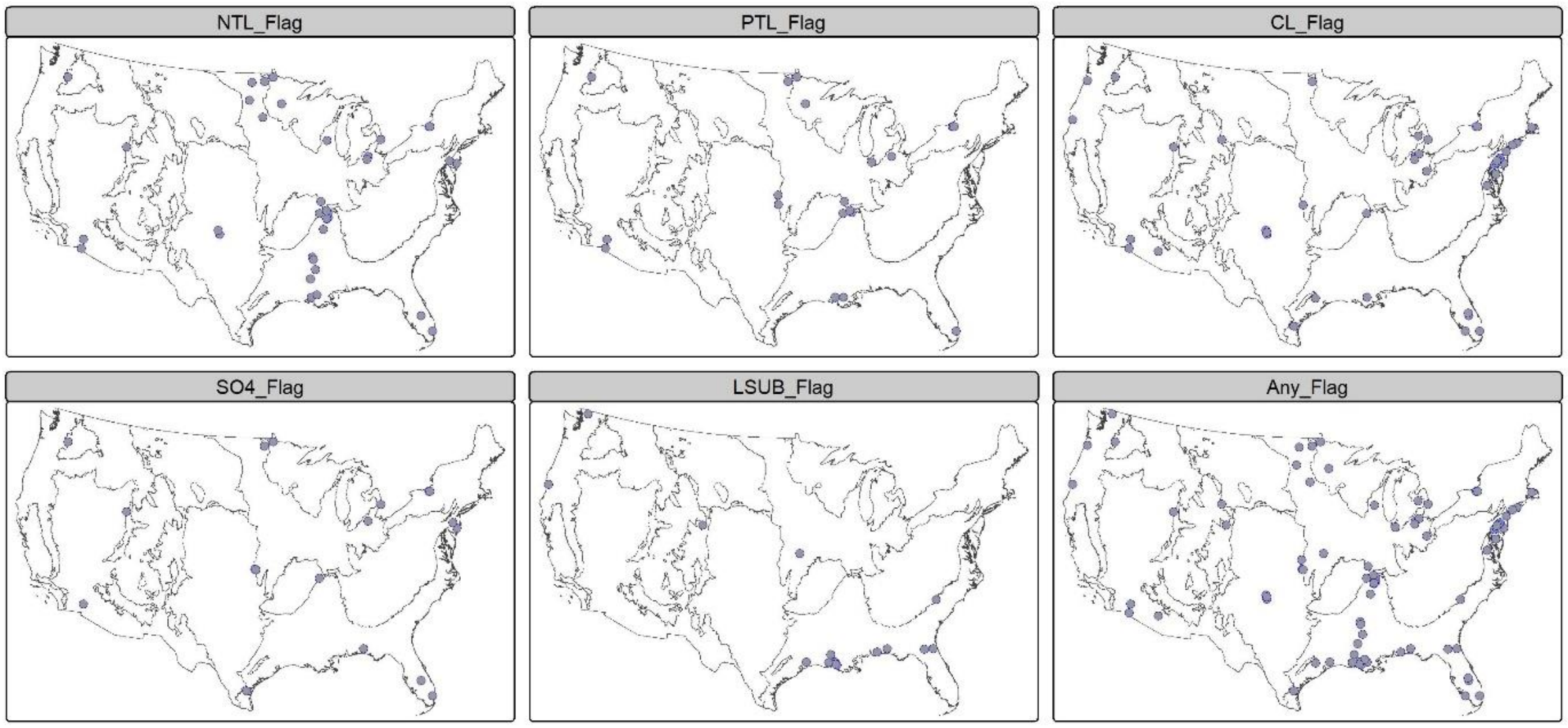

Figure S1: Sites flagged for potential extrapolation after removing anthropogenic effects from the random forest models. NTL = total nitrogen; PTL = total phosphorus; CL = chloride; SO4 = sulfate; LSUB = substrate diameter, Any = sites that were flagged for extrapolation and removed from the analysis. The number of sites removed is as follows: CPL = 40 (18%); NAP = 2 (1%); NPL = 1 (1%); SAP = 1 (<1%); SPL = 6 (3%); TPL = 13 (6%); UMW=3 (1%); WMT = 2(1%) and XER = 5 (3%).

Table S3. Variance inflation factors (VIF) for predictor variables used for joint species distribution models. In general, VIF >5 indicates a strong correlation between variables while values VIF < 3 indicate low correlation between values. Bold font = VIF >3; NTL = Total Nitrogen, PTL = Total Phosphorus, CL = Chloride, SO4 = Sulfate, SUBD = Substrate Diameter, RPDI = Riparian Disturbance index, MSAT = Mean Summer Air Temperature, PRCP = Total Annual Precipitation.

	NTL (ug/L)	PTL (ug/L)	CL (mg/L)	SO4 (mg/L)	SUB (mm)	RPDI	MSAT (DegC)	TPRCP (mm)
CPL	2.17	1.87	2.83	2.5	1.09	1.3	1.25	1.14
NAP	**3.08**	2.6	**3.34**	1.67	1.1	1.28	2.47	1.07
NPL	2.8	2.2	2.82	2.94	1.39	1.11	1.84	1.5
SAP	2.42	1.85	2.6	1.49	1.13	1.3	1.21	1.04
SPL	2.49	2.57	2.65	2.12	1.22	1.17	1.63	1.28
TPL	1.57	1.35	1.22	1.45	1.11	1.11	1.52	1.5
UMW	2.37	1.92	**3.13**	2.57	1.09	1.46	**4.25**	1.15
WMT	1.79	1.6	2.22	1.86	1.25	1.34	1.47	1.29
XER	2.28	1.77	**4.1**	**3.23**	1.29	1.19	1.69	1.44

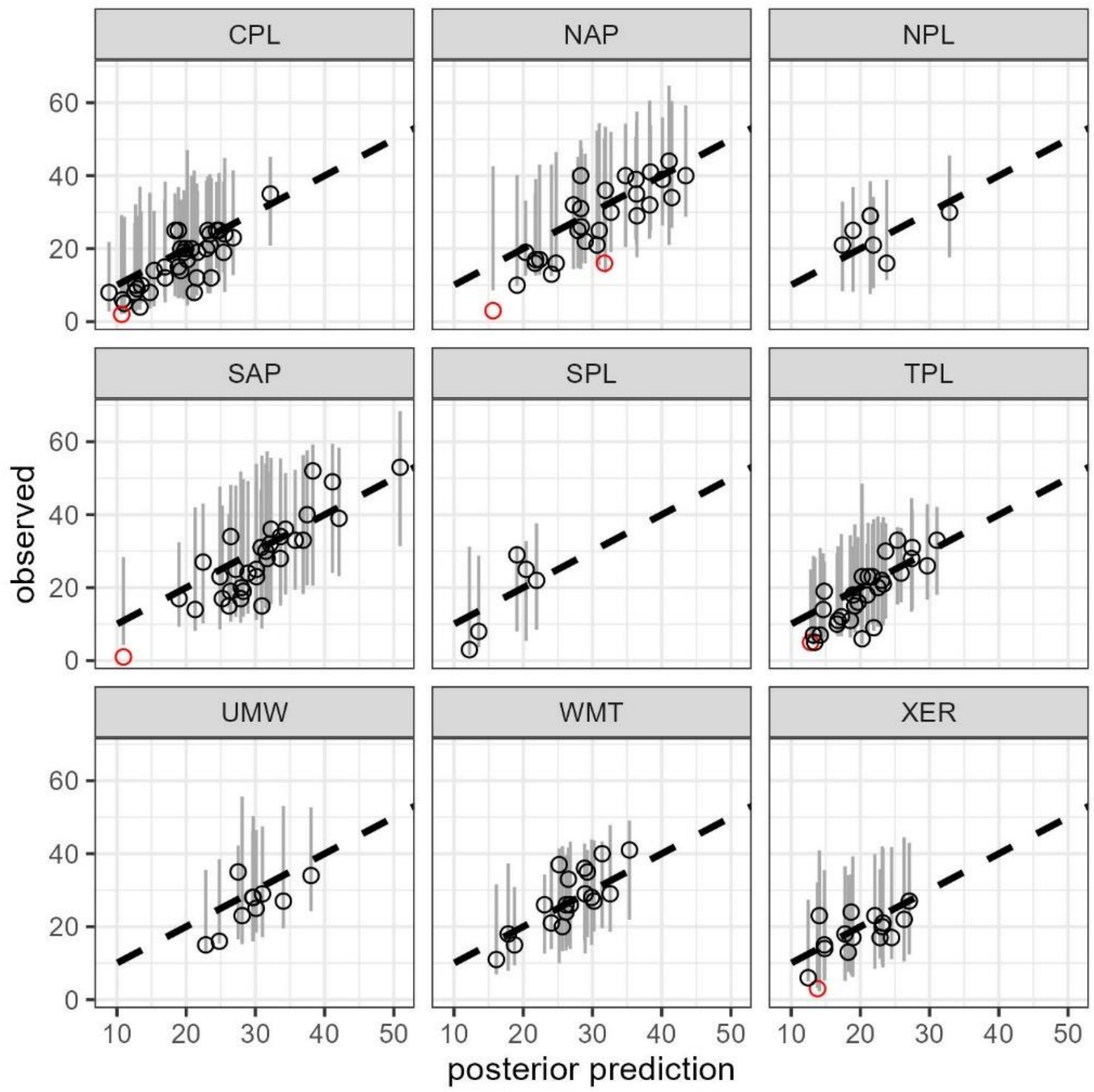

Figure S2. Validation of JSDM using revisit surveys. As part of NRSA, a subsample of sites are resurveyed. We used these data to provide validation of the predictive capabilities of JSDMs. We found that the posterior distribution of JSDM predictions typically contained the observed genus richness values. Black points indicate that the observed genus richness is within the predicted posterior distribution; Red points indicate that the observed genus richness is outside the predicted posterior distribution; Dotted line is 1:1 between observed and posterior mean richness and grey lines represent the distribution of the posterior distribution.

Table S4. Regional means of present-day (PD) and hindcasted (HC) values for each environmental gradient. Parentheses are 10th and 90th quantiles of observed hindcasted values. NTL = Total Nitrogen, PTL = Total Phosphorus, CL = Chloride, SO4 = Sulfate, SUBD = Substrate Diameter, RPDI = Riparian Disturbance index, MSAT = Mean Summer Air Temperature, PRCP = Total Annual Precipitation.

	NTL (ug/L)		PTL (ug/L)		CL (mg/L)		SO4 (mg/L)		SUB (mm)		RPDI		MSAT (DegC)		TPRCP (mm)	
	PD	HC	PD	HC	PD	HC	PD	HC	PD	HC	PD	HC	PD	HC	PD	HC
CPL	702.5 (299.2-2027.2)	380 (315.4-566.4)	77.82 (23.07-234.27)	48.67 (25.67-77.47)	8.7 (2.89-68.94)	3.7 (2.92-7.65)	6.3 (1.08-34.55)	3.29 (1.82-11.02)	0.35 (0.01-2.73)	3.32 (0.7-14.32)	0.44 (0.03-1.58)	0.33 (0.03-0.33)	27.49 (25.63-28.54)	27.02 (24.69-27.84)	1623.25 (1178.37-1990.68)	1219.49 (1015.53-1664.18)
NAP	422 (187-1301)	286 (188-327)	23.62 (7.25-71.81)	17.76 (10.04-27.89)	16.58 (0.46-72.61)	1.92 (0.3-3.82)	5.57 (1.71-15.63)	2.9 (1.74-6.07)	12.09 (0.09-122.43)	14.36 (3.4-47.45)	0.65 (0-2.02)	0.33 (0-0.33)	21.49 (18.99-23.67)	20.41 (18.31-21.75)	1299.05 (1106.82-1629.65)	1089.44 (949.5-1234.19)
NPL	881.5 (265.9-2193.5)	462.75 (216-694)	85.89 (18.01-366.21)	67.33 (38.47-85.9)	12.09 (2.34-50.02)	10.16 (1.45-19.62)	486.24 (18.73-1340.1)	172.31 (9.75-517.88)	0.51 (0.01-18.45)	3.1 (1.19-29.75)	1.27 (0.38-2.08)	0.33 (0.33-0.33)	20.52 (18.34-22.49)	21.19 (19.1-23.77)	437.42 (320.65-632.12)	361.68 (282.17-428.71)
SAP	556 (173-1876)	284 (158-350)	29.26 (9.83-128.25)	24.3 (16.41-45.85)	5.82 (1.34-47.53)	2.7 (1.08-3.82)	7.47 (1.55-80.36)	3.88 (1.88-8.95)	16.13 (0.35-206.69)	14.32 (6.1-57.76)	0.77 (0.05-2.29)	0.33 (0.05-0.33)	24.76 (22.09-26.68)	24.05 (21.37-26.37)	1476.55 (1108.36-1809.43)	1110.8 (920.87-1283.9)
SPL	1165 (438.5-2961)	405.25 (301.75-627)	143.62 (26.05-774.69)	56.88 (37.68-76.58)	29.21 (4.51-358.38)	10.47 (4.29-29.42)	86.1 (7.94-898.01)	45.6 (8.5-334.85)	0.35 (0.02-19.61)	3.18 (0.92-12.68)	1.02 (0.21-2.29)	0.33 (0.21-0.33)	25.84 (21.9-29.66)	26.17 (22.03-28.92)	616.17 (393.76-1051.75)	534.19 (312.86-977.73)
TPL	1868 (647.6-9147.8)	418 (330.6-578)	165.08 (54.39-444.97)	50.96 (40.55-63)	18.29 (7.1-62.09)	5.7 (3.7-10.38)	32.99 (11.93-256.93)	13.48 (5.28-105.55)	0.35 (0.01-16.28)	7.79 (2.51-18.96)	0.83 (0.13-2.29)	0.33 (0.13-0.33)	23.36 (21.05-25.28)	23.63 (21.3-25.38)	1004.99 (678.76-1395.29)	775.15 (537.81-922.38)
UMW	1188 (352.2-5320)	371 (289.6-511.49)	57.4 (16.93-166.88)	36.03 (17.9-53.25)	9.98 (0.76-29.9)	2.72 (0.3-5.21)	9.28 (1.12-40.68)	3.65 (1.34-8.36)	0.35 (0.01-12.6)	6.1 (2.19-27.43)	0.62 (0-2.1)	0.33 (0-0.33)	20.84 (18.13-22.56)	20.98 (18.42-22.38)	961.61 (801.29-1395.23)	799.34 (623.5-877.62)
WMT	133 (50-385.6)	133 (66-235.1)	22.12 (6.97-82.29)	21.14 (9.3-53.81)	0.92 (0.12-9.14)	0.52 (0.16-3.13)	2.97 (0.47-24.57)	3.2 (0.86-15.04)	38.14 (0.97-192.14)	38.17 (6.1-99.8)	0.48 (0-1.73)	0.33 (0-0.33)	18.08 (13.97-22.33)	16.96 (12.57-21.33)	648.86 (349.11-1670.25)	688.27 (386.2-1778.26)
XER	333 (107.2-1245.6)	253 (133.8-418)	53.87 (14.3-240.71)	42.76 (15.83-84.65)	5.23 (0.79-132.36)	3.17 (0.68-20.77)	25.08 (1.54-324.3)	20.85 (1.93-112.43)	2.18 (0.02-66.09)	10.78 (2.1-44.24)	1.14 (0.11-2.54)	0.33 (0.11-0.33)	22.76 (18.81-29.64)	21.33 (17.46-26.95)	287.58 (170.89-511.45)	337.66 (223.14-702.18)

References

Clark, C. M., J. Phelan, P. Doraiswamy, J. Buckley, J. C. Cajka, R. L. Dennis, J. Lynch, C. G. Nolte, and T. L. Spero. 2018. Atmospheric deposition and exceedances of critical loads from 1800−2025 for the conterminous United States. Ecological Applications **28**:978-1002.

Fakhraei, H., C. T. Driscoll, J. R. Renfro, M. A. Kulp, T. F. Blett, P. F. Brewer, and J. S. Schwartz. 2016. Critical loads and exceedances for nitrogen and sulfur atmospheric deposition in Great Smoky Mountains National Park, United States. Ecosphere **7**:e01466.

Granat, L., H. Rodhe, and R. O. Hallberg. 1976. The Global Sulphur Cycle. Ecological Bulletins:89-134.

Hill, R. A., M. H. Weber, S. G. Leibowitz, A. R. Olsen, and D. J. Thornbrugh. 2016. The Stream-Catchment (StreamCat) Dataset: A Database of Watershed Metrics for the Conterminous United States. JAWRA Journal of the American Water Resources Association **52**:120-128.

Sabo, R. D., C. M. Clark, J. Bash, D. Sobota, E. Cooter, J. P. Dobrowolski, B. Z. Houlton, A. Rea, D. Schwede, S. L. Morford, and J. E. Compton. 2019. Decadal Shift in Nitrogen Inputs and Fluxes Across the Contiguous United States: 2002–2012. Journal of Geophysical Research: Biogeosciences **124**:3104-3124.

Sabo, R. D., C. M. Clark, D. A. Gibbs, G. S. Metson, M. J. Todd, S. D. LeDuc, D. Greiner, M. M. Fry, R. Polinsky, Q. Yang, H. Tian, and J. E. Compton. 2021. Phosphorus Inventory for the Conterminous United States (2002–2012). Journal of Geophysical Research: Biogeosciences **126**:e2020JG005684.

Sabo, R. D., B. Pickard, J. Lin, B. Washington, C. M. Clark, J. E. Compton, M. Pennino, B. Bierwagen, S. D. LeDuc, J. N. Carleton, M. Weber, M. Fry, R. Hill, S. Paulsen, A. Herlihy, and J. L. Stoddard. 2023. Comparing Drivers of Spatial Variability in U.S. Lake and Stream Phosphorus Concentrations. Journal of Geophysical Research: Biogeosciences **128**:e2022JG007227.
